# Push-Pull OPEs in Blue-Light Anticancer Photodynamic Therapy

**DOI:** 10.3390/molecules30112310

**Published:** 2025-05-24

**Authors:** Ana Lameiro, Chiara M. A. Gangemi, Aurora Mancuso, Paola Maria Bonaccorsi, Maria Letizia Di Pietro, Silvia Gómez-Pastor, Fausto Puntoriero, Francisco Sanz-Rodríguez, Anna Barattucci

**Affiliations:** 1 Departamento de Biología, Facultad de Ciencias, Universidad Autónoma de Madrid, 28049 Madrid, Spain; ana.lameiro@estudiante.uam.es (A.L.); silvia.gomezp@uam.es (S.G.-P.); 2 Dipartimento di Scienze Chimiche, Biologiche, Farmaceutiche ed Ambientali (ChiBioFarAm), Università degli Studi di Messina, 98166 Messina, Italy; chiaramariaantonietta.gangemi@unime.it (C.M.A.G.); mancusoaurora@gmail.com (A.M.); paola.bonaccorsi@unime.it (P.M.B.); maria.dipietro@unime.it (M.L.D.P.); fausto.puntoriero@unime.it (F.P.)

**Keywords:** oligophenylene ethynylene, internal charge transfer, push-pull chromophores, blue-light photodynamic therapy

## Abstract

Photodynamic therapy (PDT) is a minimally invasive technique—used for the local eradication of neoplastic cells—that exploits the interaction of light, oxygen, and a photo-responsive drug called photosensitizer (PS) for the local generation of lethal ROS. Push-pull chromophores, that bear electron donor (D) and acceptor (A) groups linked through a π-electron bridge, are characterized by a non-homogeneous charge distribution in their excited state, with charge transfer from one extremity of the chain to the other one (Internal Charge Transfer—ICT). This phenomenon has a direct impact on the photophysical features of the push-pull compounds, as the bathochromic shift of the emission maxima and intersystem crossing (ISC) of the excited state are directly connected with the production of reactive oxygen species (ROS). In continuing our research regarding the synthesis and use of oligophenylene ethynylenes (OPEs) in PDT, two new push-pull glycosyl **OPE-NOF** and **OPE-ONF**—featuring electron-donor *N,N*-dimethylamino (**N**) and dimetoxyaryl (**O**) and acceptor tetrafluoroaryl (**F**) moieties on the OPE chain—have been efficiently prepared. The interchanged position of the D groups onto the conjugated skeleton was aimed to tune and optimize the push-pull effect, while the introduction of glucoside terminations was directed to give biocompatibility and bioaffinity to the chromophores. **OPE-NOF**, **OPE-ONF**, and the synthetic intermediates were fully characterized, and their photophysical properties were investigated by using UV-Vis absorption and emission spectroscopy. **OPE-NOF** showed a strong charge-transfer character and high PDT effect on HeLa cancer cells when irradiated with non-harmful blue light, causing massive cancer cell death.

## 1. Introduction

In the last decades, Photodynamic therapy (PDT) [1] has become a promising disease treatment for its outstanding benefits such as non-invasiveness and high *spatio*-temporal control. It generally relies on three key components: a photo-activable agent, called photosensitizer (PS), light at a discrete wavelength matching with PS absorption maxima, and oxygen. PS is administrated to the sick tissue and then excited by the light source to a singlet state that undergoes, through intersystem crossing (ISC), to the long-lived excited triplet state (T1) that reacts with oxygen and other surrounding species to produce singlet oxygen (^1^O_2_) and/or reactive oxygen species (ROS), such as peroxides, superoxide anions or hydroxyl radicals [2,3]. The irreversible oxidation of the near bioactive species is the cause of acute cell stress and death. The intracellular localization of the PS and the tumor cell typology influence the necrotic and/or apoptotic cell death mechanism [4]. In designing PDT active PSs, a common strategy is to incorporate them into the chromophore halogen atoms, such as bromine (Br) and iodine (I), as well as transition metal ions from the second and third rows of the periodic table. This approach aims to enhance the intersystem crossing (ISC) process by promoting strong spin-orbit coupling (SOC) through the presence of heavy atoms, thereby improving the efficiency of ROS generation during PDT [5,6]. An effective alternative to the use of heavy metals or heavy atoms for enhancing intersystem crossing (ISC) and reactive oxygen species (ROS) generation lies in the rational design of purely organic photosensitizers. Several strategies have emerged to achieve this goal. One approach involves enhancing spin-orbit coupling (SOC) by engaging non-bonding orbitals in the excited-state transitions—for example, substituting the oxygen atom in conventional carbonyl-containing fluorophores with sulfur. Another strategy exploits conformational dynamics, where nonplanar π-conjugated chromophores induce torsional motion, facilitating ISC through twisting-induced mechanisms. Additionally, the use of thermally activated delayed fluorescence (TADF) emitters and aggregation-induced ISC (AI-ISC) mechanisms has shown promise in metal-free ROS generation. Among these, the implementation of orthogonal donor-acceptor (D–A) molecular architectures stands out as a particularly versatile and effective approach. These systems enable the formation of charge-transfer states that promote ISC via the spin-orbit charge-transfer intersystem crossing (SOCT-ISC) mechanism, offering a robust platform for next-generation organic photosensitizers [7,8]. Conjugated D-π-A systems, or push-pull chromophores, consist of electron donor (D) and acceptor (A) units that facilitate intramolecular charge transfer (ICT) upon light absorption. This results in an excited state with a non-homogeneous charge distribution, where charge transfer occurs along the chain, creating a significant dipole moment difference compared to the ground state. The HOMO-LUMO gap is influenced by this ICT state, particularly in polar solvents where the gap widens [9]. The modification of the D-π-A arrangement with various π-linkers can further tune these properties [10,11,12]. These systems are widely applied in fields like fluorescence sensing [13], optoelectronics [14,15], biomedics [16], and PS in antibacterial and cancer PDT. Incorporating D-π-A motifs can also promote ISC in the absence of heavy metals, reducing the singlet-triplet gap that is translated into a possible enhancement of the generation of reactive oxygen species (ROS) [17]. Both full and partial charge transfer mechanisms can significantly enhance ROS generation in PSs, offering a potential alternative to traditional ISC promotion methods. Oligophenylene ethynylenes (OPE) and the corresponding polyphenylene ethynylenes (PPEs) constitute a family of luminescent probes, characterized by a rigid and stable π-conjugated linear structure with an alternation of aromatic rings and triple carbon-carbon bonds [18]. The extensive conjugation of these compounds gives them peculiar photo-physical and electronic properties, making them luminescent dyes with high quantum yields. These features can be modulated by varying the number of arylene ethynylene units or by inserting different substituent groups on the aromatic rings. Thanks to their tunable and well-defined physical and chemical properties, OPEs have found applications in sensing [19], electronics [20], as antibacterials [21], as photosensitizers in PDT for their singlet oxygen and ROS production, as probes for bio-imaging and as drug-delivery systems [22]. The first research on linear push-pull OPEs comes from Meier [23], who worked on the synthesis of four series of dialkylamino-ended OPEs and on the modulation of their ICT features in dependence of (i) the A group (CN, CHO, and NO_2_) and (ii) the chain length (from one to four units). One year later, Yamaguchi [24] reported the synthesis and photophysical studies of a new series of conjugated rod-shaped OPE oligomers, bearing a variable number of MeO (D) side-substituted aryl units and terminated with CN-or a CF_3_-arenes as acceptors, and the study of the clear influence of the solvent polarity on the variation of their light-emitting features. More recently, the same group showed how substitution with a large series of A and D groups could well-ordering change the light-emitting features of these oligomers, giving rise to a series of whole-rainbow chromophores [25]. In 2017, a new class of diphenyl amino push−pull systems, among them ICT-OPEs, featuring the highly lipophilic pentafluorosulfanyl (SF_5_) group as a strong acceptor moiety, were reported by Chan [10]. These chromophores bear a strong push−pull character, a marked solvatofluorochromism, and large Stokes shifts (>100 nm). The extensive supramolecular C−H···F interactions in their crystalline state make the authors state their potential use in crystal engineering. Recently, our group has reported the synthesis of a series of symmetric glyco-ammino OPEs in which the substituents of the aryl core strongly influence their spectrophotometric features. The synergic effect of (i) the insertion of an NMe or NMe_3_^+^ group at the aromatics, (ii) the decoration at the chain extremities with sugar residues of different nature-glucose, mannose, and maltose- [26], and (iii) the different length of the OPE chain was the key for their application in different fields of biomedicine as fluorescent intracellular dyes [27], photosensitizers in photodynamic therapy [28] and for their interaction with the DNA double helix, leading to selective tumor cells death [29]. In this paper, we report the synthesis of two new glucosyl push-pull OPE chromophores, featuring one *N,N*-dimethylamino, and two methoxy functions as D groups, and a tetrafluoroaryl moiety as A at the end of the π-conjugated system. The study of the mutual influence of the position and characteristics of the three functional groups onto the OPE chain on their spectroscopic features and their application as PSs in blue-light-induced PDT are also reported.

## 2. Results and Discussion

### 2.1. Synthesis

**Scheme 1:**
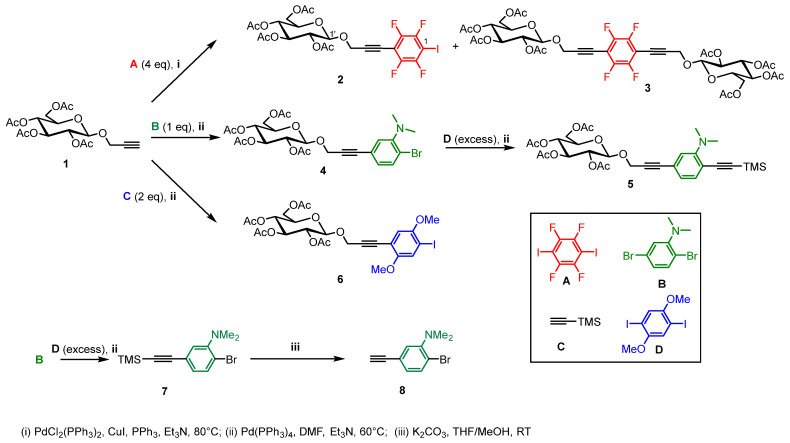
Synthetic routes to building blocks **2**–**8**.

Heck-Cassar-Sonogashira Pd mediated couplings [30] and variants were utilized for most of the synthetic steps to the new push-pull OPEs, as the simplest glycosyl-aromatic building blocks **2**, **4**, **5**, **6**, useful for the assembly of the trimeric asymmetric conjugated chain [31]. Firstly, the electron-withdrawing brick **2** was synthesized via the cross-coupling of 2,3,4,6-tetracetyl-1-propargyl-β-D-glucopyranose **1** [32] with an excess of 1,4-diiodo-2,3,5,6-tetraiodo benzene **A** (Scheme 1). Unlikely, the high reactivity of the polyfluorinated aromatic halogenide **2** in Pd-mediated cross-couplings caused, even in the presence of a large amount of **A**, the high-yield formation of the bis-glucosyl **3**, useless for our synthetic purposes.

**Scheme 2:**
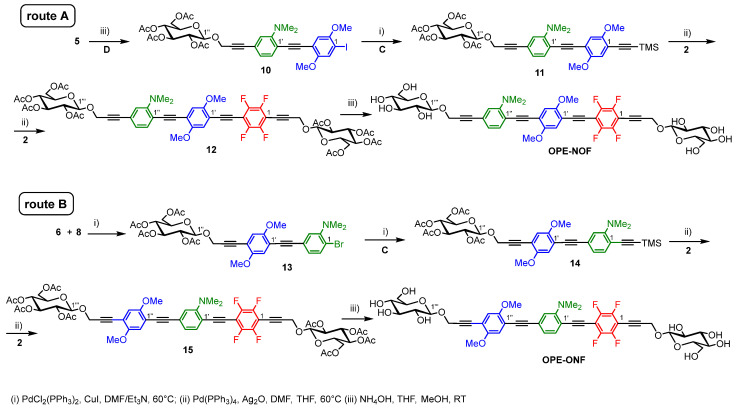
Synthetic routes to OPE-NOF and OPE-ONF.

After several attempts in different reaction conditions (see ESI, Table S1), **2** was obtained as an almost unique reaction product at 80 °C in neat Et_3_N and in the presence of the catalytic mixture Pd(PPh_3_)_2_Cl_2_, CuI and PPh_3_. Chromatographic purification furnished **2** in good yield (65%). The first electron-rich building block, the *N,N*-dimethylammino derivative **5** was synthesized via two successive copper-free Sonogashira couplings: the totally regioselective first one between *N,N*-dimethyl-2,5-dibromoaniline **B** and 2,3,4,6-tetracetyl-1-propargyl-β-D-glucopyranose **1** led to **4**, then cross-coupled with a large excess of ethynyltrimethylsilane **C** [28]. In the same reaction conditions, glycoside **1** was coupled with a twofold amount of 2,5-diiodo-1,4-dimethoxybenzene **D** to give the iodoarene **6** [27]. The regioselective cross-coupling of **B**, previously exploited for the synthesis of **4**, with ethynyltrimethylsilane **C**, afforded **7**, then deprotected with K_2_CO_3_ to 2-bromo-5-ethynyl-*N,N*-dimethylaniline **8** [33]. Scheme 2 shows the synthetic paths to the new push-pull glycosyl **OPE-NOF** (route A) and **OPE-ONF** (route B). As a first step, the synthesis of **10** was carried out through a cross-coupling of **5** with an excess of **C** in the presence of Ag_2_O and anhydrous THF as solvent, without the use of the base. The use of silver oxide is the key feature of this reaction: it is reported that [34] helps the cleavage of the C_sp_-Si bond, allowing the generation of the terminal triple bond that reacts in situ with an iodoarene. The reaction of **10** with an excess of ethynyltrimethylsylane **C** afforded **11**, then reacted in the presence of Ag_2_O with the fluoroderivative **2**, leading to **12** as a yellow powder. Deacetylation of **12** with aqueous ammonia in MeOH/THF produced quantitively final **OPE-NOF**. Several washings with Et_2_O of the solid crude, obtained from evaporation of the reaction solvents, were needed to extract the side product acetamide from the non-soluble **OPE-NOF**. **OPE-ONF** was synthesized to study the impact on ICT of the interchange of the two electron-donating groups as follows: monofunctionalized **6** was cross-coupled with **8** in the classical Sonogashira coupling conditions giving **13** in high yield [32], then reacted in a second cross-coupling with ethynyltrimethylsilane **C** to **14**.

The Ag_2_O-mediated cross-coupling of **14** with an equimolar amount of **2** gave the OPE glucoside **15** that was deacetylated in quantitative yields to **OPE-ONF** with aqueous ammonia. Apart from the final OPEs, all the intermediates were purified by column chromatography before each reaction step and fully characterized (see ESI).

### 2.2. Photophysical Properties

**Figure 1:**
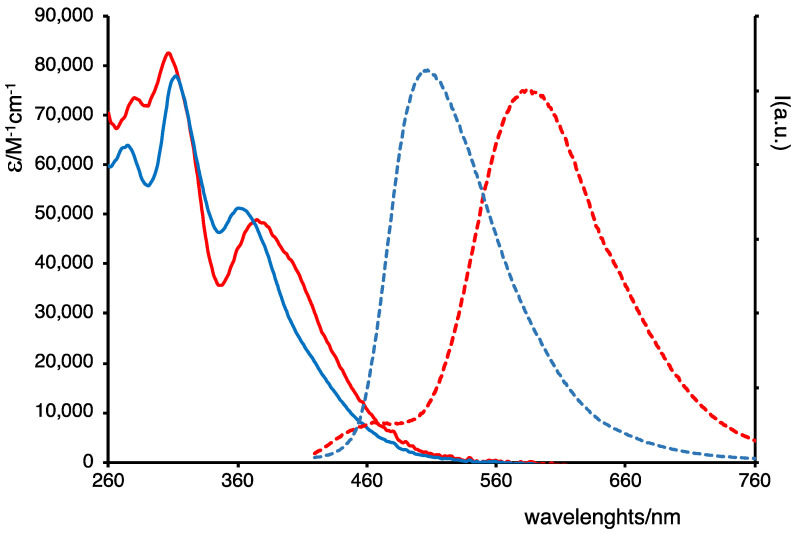
Absorption (solid line) and emission (dashed line) spectra of **OPE-ONF** (blue) and **OPE-NOF** (red) in air-equilibrated aqueous solutions.

The absorption spectra in aqueous solutions of the newly synthesized species **OPE-NOF** and **OPE-ONF** are shown in Figure 1. As can be observed for both compounds, the absorption spectra predominantly reside in the ultraviolet (UV) region of the electromagnetic spectrum, with varying contributions extending into the visible range, particularly in the initial part of the visible spectrum. More specifically, the absorption spectrum of **OPE-ONF** is characterized by two distinct absorption bands centered at approximately 270 nm and 310 nm, respectively, within the UV region. These bands can be attributed to π-π transitions, which involve the delocalized electron cloud of the aromatic backbone of the molecule. In addition to these, a broader absorption feature is observed at lower energy, around 360 nm. The absorption tail associated with this feature suggests the presence of an additional band that partially overlaps with the previous absorption transitions. Upon further inspection, this band appears to be the result of transitions exhibiting a charge transfer character. These charge transfer transitions involve a Highest Occupied Molecular Orbital (HOMO) that is predominantly influenced by the nitrogen-substituted central aromatic ring and a Lowest Unoccupied Molecular Orbital (LUMO) that is primarily influenced by the fluorine-substituted aromatic ring.

A similar absorption profile is observed for **OPE-NOF**. Although slight shifts in the spectral positions are apparent, which further corroborates the notion that the relative positioning of the aromatic rings on the linear backbone structure modulates the electronic properties of the molecule, two relatively intense bands are again observed within the UV region. The band that extends into the visible range is red-shifted by approximately 10 nm. Moreover, the shoulder at the lower energy region in the absorption spectrum of **OPE-NOF** is more pronounced and extends further into the longer wavelength region when compared to **OPE-ONF**. This behavior suggests that the transitions in **OPE-NOF** are associated with orbitals that exhibit more extensive electron delocalization compared to the transitions in **OPE-ONF**. In the case of **OPE-NOF**, the LUMO is likely to involve a more delocalized molecular orbital, which extends across the methoxy-substituted central ring and the fluorine-substituted ring. In contrast, for **OPE-ONF**, the LUMO appears to be more localized, with contributions primarily arising from the fluorine-substituted ring and the methoxy-substituted ring, albeit with different relative intensities. This analysis is further supported by the observed luminescent properties of the two species. Both **OPE-ONF** and **OPE-NOF** exhibit emission spectra, as depicted in Figure 1, which are characterized by broad and unstructured emission bands. This is a typical feature of charge-transfer state emissions, where the electronic transitions involve a significant redistribution of electron density between donor and acceptor units. Furthermore, the emission spectrum of **OPE-NOF** occurs at substantially lower energies compared to **OPE-ONF**, with emission maxima observed at 585 nm and 505 nm, respectively. This red-shifted emission of **OPE-NOF** suggests that the charge-transfer excited state is more stabilized in this compound than in **OPE-ONF**, which is further evidenced by the emission characteristics. The quantum yields of emission for **OPE-ONF** and **OPE-NOF** are 0.35 and 0.24, respectively, with excited-state lifetimes of 4.45 ns and 3.2 ns. These relatively modest quantum yields and lifetimes, in comparison with similar systems previously studied [26,27,28], indicate the possible presence of an additional non-radiative deactivation pathway. One plausible mechanism is the population of a triplet state, which is more readily populated in **OPE-NOF** due to the lower energy of the charge-transfer excited state in this compound. It is well established in the literature that triplet states in organic compounds can facilitate the generation of singlet oxygen or, more generally ROS, through mechanisms such as energy transfer or intersystem crossing. To validate this hypothesis further, we investigated the ability of these newly synthesized species to produce ROS upon exposure to low-energy blue light irradiation. The results of these experiments demonstrated that both **OPE-ONF** and **OPE-NOF** are indeed capable of generating ROS, with quantum yields of 0.19 and 0.30, respectively, see data in ESI, Figure S24. To assess whether the ROS generated upon irradiation involved predominantly singlet oxygen rather than other reactive species, we investigated the phosphorescence emission of singlet oxygen in the near-infrared region (1280 nm). Under the experimental conditions employed, no detectable singlet oxygen luminescence was observed for any of the investigated compounds. As a positive control to validate the experimental setup, singlet oxygen emission was successfully recorded, under identical conditions, using tetraphenylporphyrin (TPP), a well-known singlet oxygen sensitizer (see ESI, Figure S23).

### 2.3. Biological Study

#### 2.3.1. Dark Toxicity

The potential of **OPE-NOF** and **OPE-ONF** as photosensitizers was evaluated using HeLa cells as an in vitro model of cancer cells. The cytotoxicity of both compounds was assessed using the MTT assay to determine their effects in the absence of light. Different concentrations of **OPE-ONF** and **OPE-NOF** (ranging from 10^−6^ M to 2 × 10^−5^ M) were administered to cell cultures in the dark for 18 h. Cell survival rates were measured 24 h post-treatment. Table 1 shows no significant changes in cell viability at concentrations of 10^−6^ M, 2.5 × 10^−6^ M, and 10^−5^ M, as the results were comparable to those of the control. At the highest concentration tested (2 × 10^−5^ M), slight toxicity was observed for the **OPE-ONF** (3.86%), while the **OPE-NOF** exhibited a more pronounced cytotoxicity (13.95%). The toxicity associated with **OPE-ONF** treatment can likely be attributed to the solvent (DMSO), as cells treated with DMSO alone showed similar toxicity levels (5.4%) to those **OPE-ONF** treated. In contrast, the toxicity induced by **OPE-NOF** at 2 × 10^−5^ M was greater than that observed with DMSO. Based on these findings, subsequent studies were conducted using concentrations of **OPE-ONF** and **OPE-NOF** below 2 × 10^−5^ M to minimize their potential cytotoxic effects.

#### 2.3.2. Subcellular Localization

**Figure 2:**
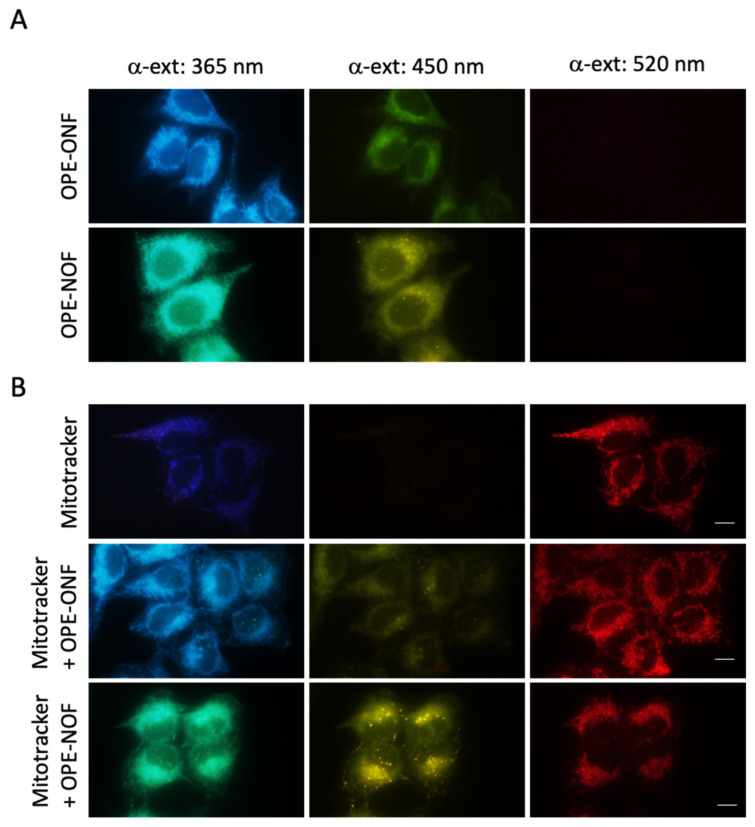
Subcellular localization of **OPE-ONF** and **OPE-NOF** in HeLa cells (**A**). **OPE-ONF** exhibited blue fluorescence under UV light excitation, while **OPE-NOF** displayed cyan fluorescence. Both compounds exhibited green fluorescence emission when excited by blue light. In control cells (**B**), mitochondria displayed autofluorescence under UV light, while the MitoTracker Red signal was visible under green light, indicating mitochondrial localization. Both **OPE-ONF** and **OPE-NOF** primarily exhibited blue and cyan fluorescence, respectively, with a localization pattern that closely matched the MitoTracker Red signal, suggesting mitochondrial targeting. Scale bar: 10 μm.

The efficiency of a photosensitizer is strongly dependent on its subcellular localization. Given the short diffusion length and brief lifetime of singlet oxygen (0.1 nm and 0.5 ms, respectively), initial oxidative reactions predominantly occur within the organelles or membrane structures where the photosensitizer is localized, as well as in surrounding cellular components, ultimately contributing to cellular photodamage [35,36]. The subcellular distribution of **OPE-ONF** and **OPE-NOF** in HeLa cells, incubated for 24 h, was assessed through their blue and green fluorescence emission under ultraviolet (UV) and blue light excitation. Fluorescent microscopy revealed significant uptake of both photosensitizers by the cells, as seen in Figure 2A,B. **OPE-ONF** exhibited blue and green emissions upon UV and blue light illumination, respectively (Figure 2A), with both signals clearly localized to the mitochondria. In contrast, **OPE-NOF** (Figure 2A) displayed both cyan and green fluorescence upon UV and blue light exposure respectively, predominantly localized in two regions: mitochondria and a reticular structure throughout the cytoplasm, likely corresponding to the endoplasmic reticulum. Furthermore, upon blue light illumination, **OPE-NOF** emitted yellow fluorescence with a slight punctate pattern, suggesting localization to cytoplasmic vesicles. It is of particular interest to emphasize that the observed coloration can be attributed to the charge transfer character inherent to both species, a property that is significantly influenced by the surrounding chemical environment. The color observed arises from the emission spectra of the compounds, which, as a result of their charge transfer nature, exhibit distinctive features. Specifically, the emission spectra of both species are notably broad, extending across a wide range of wavelengths. The presence of extended tails in the red region further highlights the nature of these emissions, indicative of transitions involving charge transfer interactions.

To validate the mitochondrial localization of both compounds, their fluorescence patterns were compared to that of the mitochondrial marker MitoTracker Red using fluorescence microscopy. As shown in Figure 2B, the subcellular localization patterns of **OPE-ONF** and **OPE-NOF** closely resembled that of MitoTracker Red. These findings suggest that both compounds are predominantly localized in the mitochondria. However, **OPE-NOF** also exhibited a punctate distribution and a reticular pattern throughout the cytoplasm, resembling the structure of the endoplasmic reticulum.

#### 2.3.3. Photodynamic Effects of OPE-ONF and OPE-NOF

**Figure 3:**
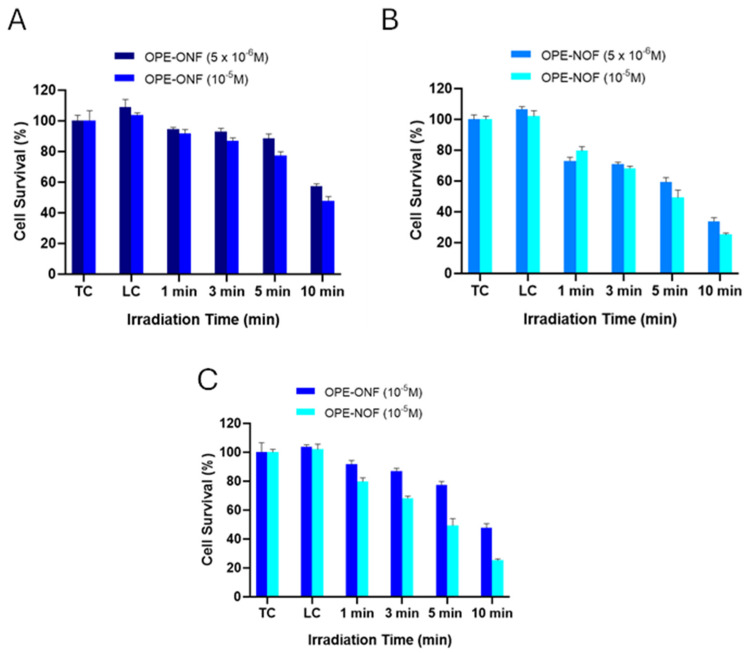
Cell survival rates following blue light PDT with (**A**) **OPE-ONF** and (**B**) **OPE-NOF**. HeLa cells were incubated for 18 h with 5 × 10^−6^ M and 10^−5^ M concentrations of both PSs. A clear phototoxic effect was observed in OPE-treated cells, which was dependent on both the concentration of the PS and the irradiation time. (**C**) Comparison of the photodynamic effects of **OPE-ONF** and **OPE-NOF** at 10^−5^ M at different irradiation times. In all cases (1–10 min of irradiation), **OPE-NOF** exhibited greater phototoxicity than **OPE-ONF**. Data points represent the mean ± SD from three independent experiments.

We next evaluated the photodynamic effects of the push-pull glucosyl-terminated OPE chromophores. HeLa cells were incubated with two different concentrations (10^−5^ M and 5 × 10^−6^ M) of **OPE-ONF** and **OPE-NOF** for 18 h, followed by irradiation with blue light for 1, 3, 5, or 10 min. Control experiments were also performed, including total control (TC), consisting of untreated cells, and light control (LC), with cells exposed to blue light but not treated with compounds. Cell viability was assessed using the MTT assay, and the results are shown in Figure 3A,B.

A clear cytotoxic effect was observed for both photosensitizers, which depended on the concentration and irradiation time. At concentrations of 5 × 10^−6^ M and 10^−5^ M, **OPE-ONF** did not induce phototoxicity higher than 20%, except after 10 min of blue light irradiation, where toxicity reached 40% and 50%, respectively, in HeLa cells. In contrast, the same concentrations of **OPE-NOF** induced more than 20% toxicity at all irradiation times tested. These findings demonstrate that, at the same concentrations, **OPE-NOF** exhibited a higher photodynamic activity compared to **OPE-ONF**. As shown in Figure 3C, the phototoxicity induced by 10^−5^ M **OPE-NOF** was consistently 14% to 39% greater than that caused by 10^−5^ M **OPE-ONF** at all irradiation times (ranging from 1 to 10 min), reaching up to 75% phototoxicity observed after 10 min of blue light exposure. Toxicity data and IC_50_ values for (A) **OPE-ONF** and (B) **OPE-NOF** are reported in ESI (Figure S26).

#### 2.3.4. Morphological Evaluation

**Figure 4:**
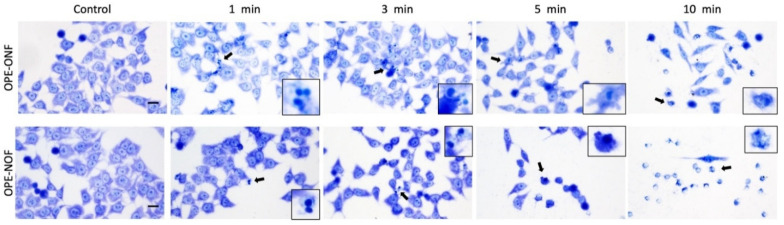
Morphological alterations induced by photodynamic treatment with **OPE-ONF** and **OPE-NOF**. HeLa Cells were incubated with 10^−5^ M **OPE-ONF** and **OPE-NOF** for 2 h, followed by irradiation with blue light at different times (1 to 10 min). After 24 h, the cells were stained with toluidine blue. The magnified images, indicated by the square in each panel, highlight cells marked with black arrowheads. Scale bar: 20 μm.

The morphological changes induced in HeLa cells by photodynamic treatments with **OPE-ONF** and **OPE-NOF** were assessed using a concentration of 10^−5^ M for both compounds, with irradiation times ranging from 1 to 10 min under blue light. Morphological evaluation was conducted 24 h post-treatment by staining with toluidine blue (TB), and the results are presented in Figure 4. As shown, cells treated with both **OPE-ONF** and **OPE-NOF** exhibited significant morphological alterations, including cytoplasmic shrinkage and loss of adhesion to the substrate, which were more pronounced following 5- and 10-min irradiation periods under blue light. Moreover, in cells treated with **OPE-NOF**, after five minutes of blue light irradiation, most of the cell population exhibited morphologies characteristic of necrotic cell death, including loss of membrane integrity and chromatin fragmentation. These alterations were also observed in cells treated with **OPE-ONF**; however, they became evident after 10 min of irradiation and affected a smaller proportion of cells compared to those treated with **OPE-NOF**. Based on these observations, it can be concluded that photodynamic treatment with both compounds at a concentration of 10^−5^ M induced necrotic cell death in HeLa cells with the effect being significantly more pronounced in cells treated with **OPE-NOF**.

**Figure 5:**
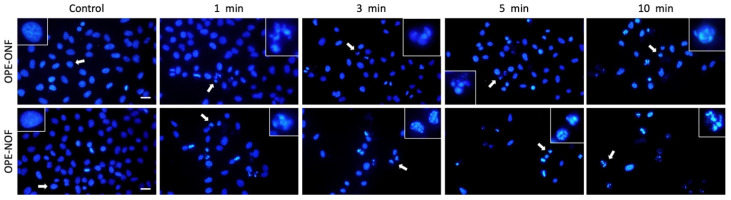
Nuclear morphological alterations induced by PDT with **OPE-ONF** and **OPE-NOF**. Control cells (control) exhibited an intact chromatin structure (white arrows in control). In contrast, all treated cells displayed characteristic features of necrotic cell death, including chromatin condensation, fragmentation of nuclear content due to membrane rupture, non-condensed degraded chromatin, and dispersed nuclear material resulting from cell lysis, all of which are typical of necrotic cells. Magnified details, indicated by white arrowheads, show these alterations in greater detail. Scale bar: 20 μm.

To validate the cell death data obtained from the TB staining assays, we conducted a nuclear morphology analysis using DAPI staining to visualize DNA in HeLa cells subjected to PDT under the same treatment conditions as in the morphological study. Well-established morphological criteria were applied to identify apoptotic and necrotic cells. The analysis of nuclear morphology performed 24 h after PDT with **OPE-ONF** and **OPE-NOF**, is presented in Figure 5. Under normal conditions, HeLa cells exhibit uniform oval nuclei with condensed chromatin, as observed in the control samples. However, when HeLa cells treated with **OPE-ONF** and **OPE-NOF** were exposed to blue light PDT, nuclear alterations indicative of necrosis were observed. These alterations included uniform chromatin condensation leading to the formation of pyknotic nuclei, as well as degraded chromatin without condensation (Figure 5). The presence of degraded chromatin without condensation, characteristic of necrotic cell death, is marked by homogeneous blue DAPI staining, resulting from random breaks in the chromatin [26]. These alterations were evident in both treatment groups (**OPE-ONF** and **OPE-NOF**) after just one minute of blue light irradiation. However, a higher proportion of cells exhibited these nuclear changes at all irradiation times following photodynamic treatment with **OPE-NOF**.

The results obtained from DAPI nuclear staining demonstrated that HeLa cells treated with 10^−5^ M **OPE-ONF** and **OPE-NOF** underwent necrotic cell death at all blue light irradiation times tested. This cell death process was dependent on the irradiation time, as longer exposure times (ranging from 5 to 10 min) resulted in an increased proportion of necrotic cells. Moreover, photodynamic treatment with **OPE-NOF** was more effective, inducing a higher percentage of necrotic cells at all irradiation times analyzed.

## 3. Experimental

### 3.1. Chemistry

Solvents were purified according to standard procedures. All the reactions were monitored by TLC on commercially available precoated plates (silica gel 60 F254), and the products were visualized with vanillin [1 g dissolved in MeOH (60 mL) and conc. H_2_SO_4_ (0.6 mL)] and UV lamp. Silica gel 60 was used for column chromatography. All reagents used were purchased from Merck (Darmstadt, Germany) and used without further purification. Proton (^1^H), carbon (^13^C), and Fluorine (^19^F) NMR spectra were recorded on a Varian 500 spectrometer (Varian, Atlanta, USA) (at 500 MHz for ^1^H; 125 MHz for ^13^C, 470.4 MHz for ^19^F) using CDCl_3_ or DMSO-d_6_ as solvent. Chemical shifts are given in parts per million (ppm) (CDCl_3_: *δ* relative to residual solvent peak for ^1^H and ^13^C δ 7.26 and 77.0 ppm respectively. DMSO-d_6_: *δ* relative to residual solvent peak for ^1^H and ^13^C δ 2.49 and 39.5 ppm respectively, *δ* relative to hexafluorobenzene peak for ^19^F δ −164.9 ppm), coupling constants (J) are given in hertz; the attributions are supported by Heteronuclear Single-Quantum Coherence (HSQC) and Correlation Spectroscopy (COSY) experiments, and given in accordance with the numeration indicated in the designed structures. Combustion analyses were carried out on a FISONS EA1108 elemental analyzer (FISONS instruments, Rochester, USA). Melting points were obtained in open capillary tubes and were uncorrected. UV/Vis absorption spectra were recorded on a Jasco V-560 spectrophotometer (Jasco Co. Ltd., Tokyo, Japan). For steady-state luminescence measurements, a Jobin Yvon-Spex Fluoromax 2 spectrofluorimeter (Horiba Jobin Yvon,. Kyoto, Japan) was used, equipped with a Hamamatsu R3896 photomultiplier (Hamamatsu City, Shizuoka, Japan). The spectra were corrected for photomultiplier response using software purchased with the fluorimeter. For the luminescence lifetimes, an Edinburgh OB 900 time-correlated single-photon-counting spectrometer (Livingston, GB) was used. As excitation sources, a Hamamatsu PLP 2 laser diode (Hamamatsu City, Shizuoka, Japan) (59 ps pulse width at 408 nm) and/or the nitrogen discharge (pulse width 2 ns at 337 nm) were employed. Emission quantum yields were determined using the optically diluted method. As luminescence quantum yield standards, we used an air-equilibrated ethanol solution of anthracene (0.2). Experimental uncertainties on the absorption and photophysical data are as follows: absorption maxima, 2 nm; molar absorption, 15%; luminescence maxima, 4 nm; luminescence lifetimes, 10%; luminescence quantum yields, 20%. The quantum yields of ROS were evaluated using indirect methods, with uric acid (UA) as the detector and methylene blue as the reference photosensitizer [29].

**Compound 2**. To a flask were added Pd(PPh_3_)_2_Cl_2_ (0.045 g, 0.065 mmol, 0.025 eq.), CuI (0.012 g, 0.06 mmol, 0.025 eq.) and PPh_3_ (0.034 g, 0.13 mmol, 0.05 eq); the flask was capped with a rubber septum and evacuated. After backfilling with N_2_, this process was repeated three times. To the flask was added dry Et_3_N (5 mL) at room temperature and the suspension was heated at 80 °C and maintained under continuous stirring. After 1 h, a solution of 2,3,4,6-tetracetyl-1-propargyl-β-D-glucopyranose **1** (1.00 g, 2.59 mmol, 1 eq.) and 1,4-diiodo-2,3,5,6-tetrafluorobenzene **A** (4.16 g, 10.36 mmol, 4 eq.) in dry Et_3_N (10 mL) were added at room temperature. The reaction mixture was heated at 80 °C and maintained under continuous stirring for 1 h, until the disappearance of **1** by TLC (hexane/EtOAc 60:40). Column chromatography was performed with hexane/EtOAc 70:30 as eluant, and compound **2** was obtained as a yellow solid (1.11 g, 1.21 mmol, 65%) and followed by **3**. TLC: *R*_f_ = 0.58 (hexane/EtOAc 60:40). Mp 124–126 °C. ^1^H NMR: *δ* 5.22 (t, *J*_2′,3′_ = *J*_3′,4′_ = 9.5, 1H, H-3′), 5.09 (t, *J*_3′,4′_ = *J*_4′,5′_ = 9.5, 1H, H-4′), 5.02 (dd, *J*_1′,2′_ = 8.5, *J*_2′,3′_ = 9.5, 1H, H-2′), 4.79 (d, *J*_1′,2′_ = 8.5, 1H, H-1′), 4.64 and 4.61 (narrow AB system, *J*_vic_ = 16.6, 2H, CH_2_C≡), 4.26 and 4.14 (split AB system, *J*_5′,6′A_ = 5.0, *J*_5′,6′_B = 2.0, *J*_6′A,6′B_ = 12.7, 2H, H_2_-6′), 3.73 (ddd, *J*_4′,5′_ = 9.5, *J*_5′,6′A_ = 5.0, *J*_5′,6′B_ = 2.0, 1H, H-5′), 2.06, 2.02, 2.00 and 1.98 (four s, 12H, 4 × CH_3_CO). ^13^C NMR: *δ* 170.5, 170.1, 169.4 and 169.3 (4 × COCH_3_), 147.0 and 146.1 (m AB system, *J*_F-C_ = 245, C-2, 3, 5, 6), 104.0 (C-4), 98.3 (C-1′), 97.0 and 72.3 (C≡C), 74.1 (C-1), 72.6 (C-3′), 72.0 (C-5′), 70.8 (C-2′), 68.1 (C-4′), 61.6 (C-6′), 56.3 (CH_2_C≡), 20.6 and 20.5 (4 × CH_3_CO). Anal. Calcd for C_23_H_21_F_4_IO_10_ (660.30): C, 41.84; H, 3.21. Found: C, 41.96 H, 3.21.

**Compound 3**: ^1^H NMR: *δ* 5.25 (t, *J*_2′,3′_ = *J*_3′,4′_ = 9.5, 2H, 2 × H-3′), 5.12 (t, *J*_3′,4′_ = *J*_4′,5′_ = 9.5, 2H, 2 × H-4′), 5.02 (dd, *J*_1′,2′_ = 8.5, *J*_2′,3′_ = 9.5, 2H, 2 × H-2′), 4.75 (d, *J*_1′,2′_ = 8.5, 2H, 2 × H-1′), 4.46 (s, 4H, 2 × CH_2_C≡), 4.28 and 4.16 (split AB system, *J*_5′,6′A_ = 4.4, *J*_5′,6′B_ = 1.7, *J*_6′A,6′B_ = 12.3, 4H, 2 × H_2_-6′), 3.78–3.75 (m, 2H, 2 × H-5′), 2.10, 2.07, 2.03 and 2.01 (four s, 24H, 8 × CH_3_CO).

**Compound 10**. To a flask Pd(PPh_3_)_4_ (0.15 g, 0.128 mmol, 0.1 eq.), Ag_2_O (0.30 g, 1.28 mmol, 1 eq.), **5** (0.77 g, 1.28 mmol, 1 eq.) and 1,4-iodo-2,5-dimethoxybenzene **D** (1.00 g, 2.56 mmol, 2 eq.) were added; the flask was capped with a rubber septum and evacuated. After backfilling with N_2_, this process was repeated three times. To the flask were added dry DMF (15.0 mL) and dry THF (7.5 mL). The reaction mixture was heated at 70 °C for 1 h until the disappearance of the starting compound **5** by TLC (hexane/EtOAc 60:40). Solvents were removed in vacuo and the solid residue was dissolved in CH_2_Cl_2_ and filtered on celite. After evaporation of the solvents, the excess of unreacted **C** was separated through precipitation from methanol. The crude obtained from the evaporation of mother liquors was purified by column chromatography on silica gel using hexane/EtOAc (70:30) as eluent to give compound **10** as a yellow solid (0.58 g, 0.73 mmol, 57%).

TLC: *R*_f_ = 0.52 (hexane/EtOAc 60:40). Mp 105–107 °C. ^1^H NMR: *δ* 7.44 (d, *J*_5′,6′_ = 7.8, 1H, H-6′), 7.29 (s, 1H, H-6), 6.95–6.92 (m, 2H, H-3′, 5′), 6.92 (s, 1H, H-3), 5.27 (t, *J*_2″,3″_ = *J*_3″,4″_ = 10.0, 1H, H-3″), 5.11 (t, *J*_3″,4″_ = *J*_4″,5″_ = 10.0, 1H, H-4″), 5.04 (dd, *J*_1″,2″_ = 8.8, *J*_2″,3″_ = 10.0, 1H, H-2″), 4.84 (d, *J*_1″,2″_ = 8.8, 1H, H-1″), 4.59 (narrow AB system, *J*_vic_ = 16.0, 2H, CH_2_C≡), 4.28 and 4.17 (split AB system, *J*_5″,6″A_ = 4.9, *J*_5″,6″B_ = 2.5, *J*_6″A,6″B_ = 12.7, 2H, H_2_-6″), 3.85 and 3.84 (two s, 6H, 2 × OCH_3_), 3.76 (ddd, *J*_4″,5″_ = 10.0, *J*_5″,6″A_ = 4.9, *J*_5″,6″B_ = 2.5, 1H, H-5″), 3.02 (s, 6H, [N(CH_3_)_2_]), 2.08, 2.05, 2.02 and 2.01 (four s, 12H, 4 × CH_3_CO). ^13^C NMR: *δ* 170.5, 170.1, 169.3 and 169.2 (4 × CO), 154.6, 154.2 and 152.2 (C-2, C-5, C-2′), 134.2 (C-6′), 123.4 (C-4′), 122.6 (C-5′), 122.0 (C-6), 119.9 (C-3′), 115.3 (C-1′), 114.4 (C-3), 113.3 (C-4), 98.3 (C-1″), 93.3, 92.4, 86.5 and 84.4 (2 × C≡C), 87.2 (C-1), 72.7 (C-3″), 71.8 (C-5″), 71.0 (C-2″), 68.2 (C-4″), 61.7 (C-6″), 56.9, 56.8 and 56.4 (CH_2_C≡ and 2 × OCH_3_), 43.2 [N(CH_3_)_2_], 20.6, 20.5 and 20.4 (4 × CH_3_CO). Anal. Calcd for C_35_H_38_INO_12_ (791.58): C, 53.11; H, 4.84; N, 1.77. Found: C, 52.95; H, 4.85; N, 1.77.

**Compound 11**. To a flask were added Pd(PPh_3_)_2_Cl_2_ (11 mg, 0.016 mmol, 0.025 eq.), CuI (3 mg, 0.016 mmol, 0.025 eq.), and **10** (0.50 g, 0.63 mmol, 1 eq); the flask was capped with a rubber septum and evacuated. After backfilling with N_2_, this process was repeated three times. To the flask was added dry Et_3_N (4.0 mL) and dry DMF (2.0 mL) at room temperature; then commercial ethynyltrimethylsilane **C** (0.17 mL, 1.26 mmol, 2 eq.) was added. The reaction mixture was heated at 70 °C and maintained under continuous stirring for 2 h, until the disappearance of **10** by TLC (hexane/EtOAc 60:40). The resulting crude was filtered on Celite and column chromatography was performed with hexane/EtOAc 70:30 as eluant. Compound **11** was obtained as a yellow solid (0.35 g, 0.46 mmol, 73%). TLC: *R*_f_ = 0.48 (hexane/EtOAc 60:40). Mp 110–112 °C. ^1^H NMR: *δ* 7.43 (d, *J*_5′,6′_ = 7.8, 1H, H-6′), 6.95–6.93 (m, 4H, H-3, 6, 3′, 5′), 5.27 (t, *J*_2″,3″_ = *J*_3″,4″_ = 9.8, 1H, H-3″), 5.11 (t, *J*_3″,4″_ = *J*_4″,5″_ = 9.8, 1H, H-4″), 5.03 (dd, *J*_1″,2″_ = 7.8, *J*_2″,3″_ = 9.8, 1H, H-2″), 4.83 (d, *J*_1″,2″_ = 7.8, 1H, H-1″), 4.59 (narrow AB system, *J*_vic_ = 16.0, 2H, CH_2_C≡), 4.28 and 4.15 (split AB system, *J*_5″,6″A_ = 4.4, *J*_5″,6″B_ = 3.0, *J*_6″A,6″B_ = 12.2, 2H, H_2_-6″), 3.85 and 3.84 (two s, 6H, 2 × OCH_3_), 3.75 (ddd, *J*_4″,5″_ = 9.8, *J*_5″,6″A_ = 4.4, *J*_5″,6″B_ = 3.0, 1H, H-5″), 3.01 (s, 6H, [N(CH_3_)_2_]), 2.07, 2.04, 2.01 and 2.00 (four s, 12H, 4 × CH_3_CO), 0.26 [s, 9H, Si(CH_3_)_3_]. ^13^C NMR: *δ* 170.6, 170.2, 169.4 and 169.3 (4 × CO), 154.6, 154.2 and 153.8 (C-2, C-5, C-2′), 134.3 (C-6′), 123.4 and 119.9 (C-3′, 5′), 122.7 (C-4′), 115.8 and 115.2 (C-3, 6), 115.4, 113.9 and 113.0 (C-1, 4, 1′), 98.4 (C-1″), 100.9, 100.4, 94.1, 92.8, 87.3 and 84.4 (3 × C≡C), 72.8 (C-3″), 71.9 (C-5″), 71.1 (C-2″), 68.3 (C-4″), 61.8 (C-6″), 56.9, 56.5 and 56.4 (CH_2_C≡ and 2 × OCH_3_), 43.3 [N(CH_3_)_2_], 20.7 and 20.5 (4 × CH_3_CO), -0.04 [Si(CH_3_)_3_]. Anal. Calcd for C_40_H_47_NO_12_Si (761.89): C, 63.06; H, 6.22; N, 1.84. Found: C, 63.02; H, 6.21; N, 1.84.

**Compound 12**. Compounds **11** (0.33 g, 0.44 mmol, 1 eq.) and **2** (0.29 g, 0.44 mmol, 1 eq.), Ag_2_O (0.10 g, 0.44 mmol, 1 eq.) and Pd(PPh_3_)_4_ (0.05 g, 0.044 mmol, 0.1 eq.) were suspended in dry DMF (5.0 mL) and THF (2.5 mL). The obtained mixture was heated at 70 °C and maintained under Ar atmosphere and continuous stirring for 1.5 h, until the disappearance of starting products by TLC (hexane/EtOAc 50:50). After filtration over Celite, the solvents were removed under reduced pressure, and the obtained reaction crude was subjected to silica gel column chromatography. Column chromatography was performed with hexane/EtOAc 60:40 as eluent, and compound **12** was obtained as a brilliant yellow solid (0.27 g, 0.22 mmol, 50%). TLC: *R*_f_ = 0.45 (hexane/EtOAc 50:50). Mp 126–128 °C. ^1^H NMR: *δ* 7.46 (d, *J*_5″,6″_ = 7.9, 1H, H-6″), 7.03 and 7.01 (two s, 2H, H-3′,6′), 6.98–6.96 (m, 2H, H-3″, 5″), 5.30–5.23 (m, 2H, 2 × H-3‴), 5.14–5.10 (m, 2H, 2 × H-4‴), 5.07–5.02 (m, 2H, 2 × H-2‴), 4.85–4.82 (m, 2H, 2 × H-1‴), 4.68, 4.65 and 4.60 (narrow AB system and s, *J*_vic_ = 15.9, 4H, 2 × CH_2_C≡), 4.31–4.17 (m AB system, 4H, 2 × H_2_-6‴), 3.91 and 3.90 (two s, 6H, 2 × OCH_3_), 3.78–3.73 (m, 2H, 2 × H-5‴), 3.03 [s, 6H, N(CH_3_)_2_], 2.09, 2.08, 2.06, 2.05, 2.04, 2.03 and 2.01 (seven s, 24H, 8 × CH_3_CO). ^13^C NMR: *δ* 170.7, 170.6, 170.3, 170.2, 169.5, 169.4 and 169.3 (8 x CO), 154.5, 154.4 and 153.9 (C-2′, 5′, 2″), 147.0 and 146.3 (m AB system, *J*_F-C_ = 249, C-2, 3, 5, 6), 134,5 (C-6″), 123.5 and 120.0 (C-3″,5″), 123.0 (C-4″), 115.4 and 115.2 (C-3′,6′), 115.8, 115.1 and 111.2 (C-1′, 4′, 1″), 105.9 and 103.8 (C-1,4), 100.1 and 97.4 (C≡C), 98.4 (2 × C-1‴), 95.1, 92.6, 87.2 and 84.6 (C≡C), 79.6 and 72.7 (C≡C), 72.8 (2 × C-3‴), 71.9 (2 × C-5‴), 71.0 (2 × C-2‴), 68.2 (2 × C-4‴), 61.7 (2 × C-6‴), 56.9, 56.6 and 56.3 (2 × CH_2_C≡ and 2 × OCH_3_), 43.3 [N(CH_3_)_2_], 20.8, 20.7, 20.6 and 20.4 (8 × CH_3_CO). Anal. Calcd for C_60_H_59_F_4_NO_22_ (1222.11): C, 58.97; H, 4.87; N, 1.15. Found: C, 59.05; H, 4.88; N, 1.15.

**OPE-NOF** Compound **12** (0.19 g, 0.15 mmol) was dissolved in THF/MeOH (1:1, 30 mL). To this solution, a large excess of aqueous ammonia (10 mL) was added. The reaction was maintained under continuous stirring at room temperature overnight, until the disappearance of the starting product as observed by TLC. The solvents were removed under reduced pressure and the undesired acetamide was eliminated by a series of Et_2_O washings of the obtained solid. **OPE-NOF** was obtained as a yellow solid (0.13 g, 0.15 mmol, 98%). TLC: *R*_f_ = 0.03 (CHCl_3_/MeOH 80:20). Mp 205–208 °C. ^1^H NMR (DMSO-d6): δ 7.43 (d, *J*_5″,6″_ = 8.0, 1H, H-6″), 7.21 and 7.19 (two s, 2H, H-3′,6′), 6.96–6.94 (m, 2H, H-3″,5″), 5.40 (d, *J*_vic_ = 5.0, 2H, 2 × OH), 5.32 (d, *J*_vic_ = 5.0, 2H, 2 × OH), 5.01–4.81 (m, 4H, 4 × OH), 4.83–4.51 (m, 6H, 2 × CH_2_C≡, 2 × OH), 4.35–4.30 (m, 2H, 2 × H-1‴), 3.87 and 3.86 (two s, 6H, 2 × OCH_3_), 3.71–3.41 (m, 4H, 2 × CH_2_-6‴), 3.18–2.98 (m, 14H, 2 × H-2‴-5‴, N(CH_3_)_2_). ^13^C NMR (DMSO-d6): δ 154.1, 154.0 and 153.5 (C-2′,5′,5″), 144.0–148.0 (m, C-2, 3, 5, 6), 134.5 (C-6″), 122.1 (Cq), 123.1, 122.6, 115.2 and 115.1 (C-3′,6′,3″,5″), 115.0, 113.3, 110.0 and 101.1 (Cq), 101.2 (2 × C-1‴), 95.2, 92.4, 87.2, and 85.6 (C≡C), 77.2, 77.0, 76.7, 73.3. 73.2, 70.5 and 70.0 (2 × C-2‴-5‴), 61.1 (2 × C-6‴), 56.3 and 56.2 (2 × OCH_3_), 55.7 and 55.4 (2 × CH_2_C), 42.6 [N(CH_3_)_2_]. ^19^F NMR (DMSO-d6): δ from −140.7 to −139.8 (m, F-2,3,5,6). Anal. Calcd for C_44_H_43_F_4_NO_14_ (885.81): C, 59.66; H, 4.89; N, 1.58. Found: C, 59.83; H, 4.88; N. 1.58.

**Compound 14**. To a flask were added Pd(PPh_3_)_2_Cl_2_ (21 mg, 0.03 mmol, 0.025 eq.), CuI (21 mg, 0.03 mmol, 0.025 eq.), and PPh_3_ (16 mg, 0.06 mmol, 0.05 eq); the flask was capped with a rubber septum and evacuated. After backfilling with N_2_, this process was repeated three times. To the flask was added dry Et_3_N (5.2 mL) at room temperature and the suspension was heated at 80 °C, and maintained under continuous stirring. After 1 h, a solution of compound **13** (0.80 g, 1.08 mmol, 1eq.) in dry DMF (2.6 mL) was added at room temperature and the mixture was stirred to uniform; then commercial ethynyltrimethylsilane **C** (0.92 mL, 6.48 mmol, 6 eq.) was slowly added. The reaction mixture was heated at 80 °C and maintained under continuous stirring for 5.5 h, until the disappearance of **13** by TLC (hexane/EtOAc 60:40). Column chromatography was performed with hexane/EtOAc 70:30 as eluant, and compound **14** was obtained as a yellow solid (0.58 g, 0.76 mmol, 70%). TLC: *R*_f_ 0.52 (hexane/EtOAc 50:50). Mp 127–128 °C ^1^H NMR: *δ* 7.52 (d, *J*_5,6_ = 7.8, 1H, H-6), 7.03 and 6.94 (two s, 2H, H-3′-6′), 7.03–7.01 (m, 2H, H-3,5), 5.27 (t, *J*_2″,3″_ = *J*_3″,4″_ = 9.5, 1H, H-3″), 5.12 (t, *J*_3″,4″_ = *J*_4″,5″_ = 9.5, 1H, H-4″), 5.05 (dd, *J*_1″,2″_ = 8.0, *J*_2″,3″_ = 9.5, 1H, H-2″), 4.91 (d, *J*_1″,2″_ = 8.0, 1H, H-1″), 4.65 (s, 2H, CH_2_C≡), 4.28 and 4.17 (split AB system, *J*_5″,6″A_ = 2.3, *J*_5″,6″B_ =4.7, *J*_6″A,6″B_ = 12.4, 2H, H_2_-6″), 3.88 and 3.87 (two s, 6H, 2 × OCH_3_), 3.78–3.74 (ddd, *J*_4″,5″_ = 9.5, *J*_5″,6″A_ = 2.3, *J*_5″,6″B_ =4.7, 1H, H-5″), 2.97 [s, 6H, N(CH_3_)_2_], 2.13, 2.09, 2.08 and 2.06 (four s, 12H, 4 × CH_3_CO), 0.26 [s, 9H, Si(CH_3_)_3_]. ^13^C NMR: δ 170.7, 170.3, 169.5 and 169.4 (4 × CO), 154.9, 154.1 and 153.8 (C-2 e C-2′,5′), 134.8 (C-6), 123.4 (C-5), 119.9 (C-3), 123.7, 115.0, 113.8 and 112.3 (C-1,4,1′,4′), 115.7 and 115.6 (C3′,6′), 104.3, 101.6, 95.4, 89.0, 86.4 and 83.5 (3 × C≡C), 98.3 (C-1″), 72.9 (C-3″), 71.9 (C-5″), 71.1 (C-2″), 68.4 (C-4″), 61.8 (C-6″), 57.1 (CH_2_C≡), 56.5 and 56.3 (2 × OCH_3_), 43.2 [N(CH_3_)_2_], 20.7 and 20.6 (4 × CH_3_CO), −0.09 [Si(CH_3_)_3_]. Anal Calcd for C_40_H_47_NO_12_Si (761,90): C, 63.06; H, 6.22; N, 1.84. Found C, 63.01; H, 6.21; N, 1.84.

**Compound 15**. Compounds **14** (0.54 g, 0.7 mmol, 1 eq.) and **2** (0.46 g, 0.7 mmol, 1 eq.), Ag_2_O (0.16 g, 0.7 mmol, 1 eq.) and Pd(PPh_3_)_4_ (0.08 g, 0.07 mmol, 0.1 eq.) were suspended in dry DMF (8.0 mL) and THF (4.0 mL). The obtained mixture was heated at 70 °C and maintained under Ar atmosphere and continuous stirring for 1.5h, until the disappearance of starting products by TLC (hexane/EtOAc 50:50). After filtration over Celite, the solvents were removed under reduced pressure, and the obtained reaction crude was subjected to silica gel column chromatography, performed with hexane/EtOAc 60:40 as eluant. Compound **15** was obtained as a brilliant yellow solid (0.45 g, 0.37 mmol, 53%). TLC: *R*f =0.45 (hexane/EtOAc 50:50). Mp 128–130 °C. ^1^H NMR: *δ* 7.49 (d, *J*_5′,6′_ = 7.8, 1H, H-6′), 7.09–7.07 (m, 2H, H-3′, 5′), 7.02 and 6.95 (two s, 2H, H-3″,6″), 5.30–5.22 (m, 2H, 2 × H-3‴), 5.14–5.10 (m, 2H, 2 × H-4‴), 5.08–5.03 (m, 2H, 2 × H-2‴), 4.91 and 4.83 (two d, *J*_1″′,2″′_ = 7.8, 2H, 2 × H-1‴), 4.72–4.64 (m, 4H, 2 x CH_2_C≡), 4.32–4.17 (m, 4H, 2 × H_2_-6‴), 3.89 and 3.88 (two s, 6H, 2 × OCH_3_), 3.79–3.79 (m, 2H, 2 × H-5‴), 3.04 [s, 6H, N(CH_3_)_2_], 2.09, 2.08, 2.06, 2.05, 2.04, 2.03, 2.02, and 2.01 (eight s, 24H, 8 × CH_3_CO). 13C NMR: δ 170.9, 170.8, 170.6, 170.5, 169.8, 169.7, and 169.6 (8 × CO), 155.2, 154.3 and 154.2 (C-2′, 2″, 5″), 147.2 and 146.3 (m AB system, *J*_F-C_ = 252, C-2, 3, 5, 6), 135.2 (C-6′), 125.6 (C-4′), 123.6 and 120.2 (C-3′,5′), 116.0 and 115.8 (C-3″,6″), 113.8 and 112.8 (C-1′, 1″,4″), 106.5 and 103.2 C-1, 4), 103.6 and 97.6 (C≡C-ArF), 98.3 (2 × C-1‴), 95.1, 89.2, 87.3 and 83.4 (2 × C≡C), 81.1 and 72.7 (C≡C-ArF), 72.8 and 72.7 (2 x C-3‴), 72.0 and 71.9 (2 × C-5‴), 71.1 and 70.9 (2 × C-2‴), 68.3 and 68.2 (2 × C-4‴), 61.8 and 61.7 (2 × C-6‴), 57.3, 57.0, 56.7 and 56.6 (2 × CH_2_C≡ and 2 × OCH_3_), 43.6 [N(CH_3_)_2_], 21.0, 20.9, 20.8 and 20.7 (8 × CH_3_CO). Anal. Calcd for C_60_H_59_F_4_NO_22_ (1222.11): C, 58.97; H, 4.87; N, 1.15. Found: C, 58.88; H, 4.87; N, 1.15.

**OPE-ONF** Compound **15** (0.15 g, 0.12 mmol) was dissolved in THF/MeOH (1:1, 25 mL). To this solution, a large excess of aqueous ammonia (9 mL) was added. The reaction was maintained under continuous stirring at room temperature overnight, until the disappearance of the starting product as observed by TLC. The solvents were removed under reduced pressure and the undesired acetamide was eliminated by a series of Et_2_O washings of the obtained solid. **OPE-ONF** was obtained as a yellow solid (0.10 g, 0.12 mmol, 98%). TLC: *R*_f_ = 0.03 (CHCl_3_/MeOH 80:20). Mp 200–202 °C. ^1^H NMR (DMSO-d6): δ 7.49 (d, *J*_5′,6′_ = 8.0, 1H, H-6′), 7.17 and 7.08 (two s, 2H, H-3″,6″), 7.08–7.03 (m, 2H, H-3′,5′), 5.19 (d, *J*_vic_ = 5.0, 2H, 2 × OH), 5.13 (d, *J*_vic_ = 5.0, 2H, 2 × OH), 4.99–4.81 (m, 4H, 4 × OH), 4.82–4.50 (m, 6H, 2 × CH_2_C≡, 2 × OH), 4.35–4.30 (m, 2H, 2 × H-1‴), 3.82 and 3.80 (two s, 6H, 2 × OCH_3_), 3.71–3.41 (m, 4H, 2 × CH_2_-6), 3.18–2.98 (m, 14H, 2 x H-2‴-5‴, N(CH_3_)_2_). ^13^C NMR (DMSO-d6): δ 154.7, 153.6 and 153.5 (C-5′, 2″, 5″), 147.5–144.8 (m, C-2, 3, 5, 6), 135.0 (C-6′), 125.1 (Cq), 122.6, 119.4, 115.8 and 115.6 (C-3′,5′,3″,6″), 112.5, 112.1, 110.9, 103.0 and 100.2 (Cq), 101.2 and 101.1 (2 × C-1‴), 94.4, 91.4, 88.0, 82.0 and 80.8 (C≡C), 77.1, 77.0, 76.7, 76.6, 73.3. 73.2, 70.1 and 70.0 (2 × C-2‴-5‴), 61.2 and 62.1 (2 × C-6‴), 56.3 and 56.2 (2 × OCH_3_), 55.8 and 55.5 (2 × CH_2_C), 42.7 [N(CH_3_)_2_]. ^19^F NMR (DMSO-d6): δ −139.4 and −139.7 (two AB m, F-2,3,5,6). Anal. Calcd for C_44_H_43_F_4_NO_14_ (885.81): C, 59.66; H, 4.89; N, 1.58. Found: C, 59.78; H, 4.88; N. 1.58.

### 3.2. Cell Culture

HeLa human cervical cancer cells (ATCC) were cultured as a monolayer in Dulbecco’s modified Eagle’s medium (DMEM) supplemented with 10% fetal calf serum (FCS), 50 units/mL penicillin, and 50 µg/mL streptomycin. The cells were maintained at 37 °C in a humidified atmosphere with 5% CO_2_, with the medium replaced daily. For photocytotoxicity assays, the cells were seeded into 24-well plates, while for fluorescence imaging, they were plated onto coverslips in 6-well plates.

### 3.3. Intracellular Localization of **OPE-ONF** and **OPE-NOF**

The cells were seeded onto coverslips in 6-well plates and allowed to grow for 48 h. Following this, the cells were incubated with 3 μM of each PS for 24 h. The samples were then washed twice with PBS and mounted onto slides with PBS. To further investigate the intracellular localization of **OPE-ONF** and **OPE-NOF**, the distribution patterns of both compounds were compared to that of mitochondria. For this purpose, cells were incubated with each PS for 24 h. Following drug treatment, the culture medium was replaced, and the cells were incubated with a MitoTracker Red probe (Invitrogen, UK) (at the concentrations recommended by the supplier) for 15 min. After incubation, the cells were washed twice with PBS and observed under a fluorescence microscope. The MitoTracker Red probe was excited at 545 nm, with emission detected at 599 nm. Fluorescence imaging was conducted using an Olympus BX61 microscope (Olympus, Münster, Germany) equipped with filter sets for UV (365 nm), blue (450–490 nm; excitation filter BP 490), and green (545 nm; excitation filter BP 545) channels. Images were captured using an Olympus DP74 digital camera.

### 3.4. Photodynamic Treatment In Vitro

HeLa cells seeded in 24-well plates were incubated for 18 h with the appropriate volume of each push-pull glucosyl-terminated OPE chromophore. After incubation, the cells were washed with PBS and then irradiated with blue light (450 nm) at an irradiance of 15.9 mW/cm^2^ at different times. Following irradiation, the medium was replaced with a fresh complete medium, and the cells were incubated in the dark for an additional 24 h. Cell viability was then assessed.

### 3.5. Viability Assay

Cell viability and phototoxicity were assessed using the MTT assay. 24 h after the appropriate treatment, 3-[4,5-dimethylthiazol-2-yl]-2,5-diphenyltetrazolium bromide (MTT) solution was added to each well at a concentration of 0.5 mg/mL, and the plates were incubated at 37 °C for 2 h. The resulting formazan crystals were then dissolved by the addition of dimethyl sulfoxide (DMSO), and absorbance was measured at 542 nm. The same procedure was performed without irradiation to assess dark toxicity.

### 3.6. Cell Morphology

The changes in cell morphology were analyzed using bright field illumination. After PDT treatment, the cells were fixed with cold methanol for 7 min. After washing three times with PBS, the cells were stained with toluidine blue (0.5 mg mL^−1^ in distilled water, 5 min). After being washed and air-dried, preparations were mounted in toluene and observed under bright field illumination.

### 3.7. DAPI Staining

At different times after treatment, cells were fixed for 20 min at 4 °C in a fixation solution composed of Formaldehyde (1:10 in PBS) and 0.1% Triton. Subsequently, they were stained with DAPI (100 μM in PBS) for 1 min. After staining, they were washed with PBS and mounted on slides with Vectashield Vibrance® (Vector Laboratories, CA, USA). The samples were then examined under a fluorescence microscope with UV excitation. Morphological criteria commonly used to identify necrotic and apoptotic cells were applied for assessment [26].

## 4. Conclusions

In summary, we have efficiently prepared two push-pull glucosyl-terminated OPE chromophores through a synthetic route that involves a series of high-yield Pd mediated cross-coupling reactions. **OPE-NOF** and **OPE-ONF** have been studied from the photophysical point of view, confirming their nature of ICT systems, and their ROS production under blue-light irradiation has been indirectly measured, resulting in 0.30 and 0.19 quantum yields respectively. The compounds were efficiently internalized by cancer cells, demonstrating excellent biocompatibility in the absence of light. While both compounds primarily localized to the mitochondria, **OPE-NOF** additionally showed distribution in an endoplasmic reticulum-like membranous structure and cytoplasmic vesicles. PDT under blue light irradiation (450 nm) resulted in a superior photodynamic effect of **OPE-NOF** compared to **OPE-ONF**. The synergy between the greater ROS production and the broader subcellular distribution of **OPE-NOF** is supposed to contribute to its higher photodynamic efficacy. Despite these differences, both treatments induced massive tumor cell death. These results open the way for the not-yet-reported use of push-pull OPEs in superficial blue-light-induced photodynamic therapy.

## Figures and Tables

**Table 1:** Survival rates (in percentages) of HeLa cells after 18 h of incubation in the dark with different concentrations of **OPE-ONF** and **OPE-NOF** compounds.

	HeLa	
[PS]	OPE-ONF	OPE-NOF
Control	100 ± 3.2	100 ± 0.88
DMSO	94.60 ± 3.26 *	94.60 ± 3.26 *
10^−6^ M	103.16 ± 5.65	101.29 ± 4.95
2.5 × 10^−6^ M	102.33 ± 1.78	100.67 ± 4.78
5 × 10^−6^ M	102.89 ± 2.00	100.69 ± 5.72
10^−5^ M	104.32 ± 2.44	100.97 ± 6.66
2 × 10^−5^ M	96.14 ± 0.97	86.05 ± 2.64

Each point corresponds to the mean value ± SD from three different experiments. * The survival values for DMSO correspond to the maximum volume used in the experiment, which corresponds to the volume added at the maximum concentration used (2 × 10^−5^ M) of each compound.

## Data Availability

The datasets generated and/or analysed during the current study are available from the corresponding authors upon reasonable request.

## Supplementary Materials

The following supporting information can be downloaded at: https://www.mdpi.com/article/10.3390/molecules30112310/s1, Table S1: reaction conditions for the synthesis of **2**; Figure S1: ^1^H-NMR spectrum of compound **2** in CDCl_3_; Figure S2: ^13^C-NMR spectrum of compound **2** in CDCl_3_; Figure S3: ^1^H-NMR spectrum of compound **3** in CDCl_3_; Figure S4: ^1^H-NMR spectrum of compound **10** in CDCl_3_; Figure S5: ^13^C-NMR spectrum of compound **10** in CDCl_3_; Figure S6: ^1^H-NMR spectrum of compound **11** in CDCl_3_; Figure S7: ^13^C-NMR spectrum of compound **11** in CDCl_3_; Figure S8: ^1^H-NMR spectrum of compound **12** in CDCl_3_; Figure S9: ^13^C-NMR spectrum of compound **12** in CDCl_3_; Figure S10: ^1^H-NMR spectrum of compound **OPE-NOF** in DMSO-d_6_; Figure S11: ^13^C-NMR spectrum of compound **OPE-NOF** in DMSO-d_6_; Figure S12: ^1^H-NMR spectrum of compound **14** in CDCl_3_; Figure S13: ^13^C-NMR spectrum of compound **14** in CDCl_3_; Figure S14: ^1^H-NMR spectrum of compound **15** in CDCl_3_; Figure S15: ^13^C-NMR spectrum of compound **15** in CDCl_3_; Figure S16: ^1^H-NMR spectrum of compound **OPE-ONF** in DMSO-d_6_; Figure S17: ^13^C-NMR spectrum of compound **OPE-ONF** in DMSO-d_6_; Figure S18: ^19^F-NMR spectrum of compound **OPE-NOF** in DMSO-d_6_; Figure S19: ^19^F-NMR spectrum of compound **OPE-ONF** in DMSO-d_6_; Figure S20: Absorption spectra of **OPE-NOF** in aqueous solution before irradiation and after 30 min, 1 h, and 1.5 h of blue light irradiation at 450 nm; Figure S21: Absorption of **OPE-ONF** in aqueous solution before irradiation and after 30 min, 1 h, and 1.5 h of blue light irradiation at 450 nm; Figure S22: Normalized emission spectra of **OPE-NOF** in aqueous solution befored and after the addition of one equivalent of acetic acid, in dichloromethane, and in acetonitrile; Figure S23: Emission spectra of **OPE-NOF**, **OPE-ONF** and **TPP** in aqueous solution upon excitation at 450 nm; Figure S24: Absorbance trend at 280 nm at the absorption maximum of uric acid upon irradiation of an aqueous solution of **OPE-ONF** and **OPE-NOF**; Figure S25: Drug and DMSO controls for HeLa cells subjected to blue light PDT with **OPE-ONF** and **OPE-NOF**; Figure S26: Toxicity data and IC_50_ values for **OPE-ONF** and **OPE-NOF** after irradiation with blue light for 0, 1, 3, 5, 10 min; Figure S27. Flow cytometry assays on **OPE- ONF** using the PE Annexin V Apoptosis Detection Kit with 7-AAD.
