# Supplementary material for: Push-Pull OPEs in Blue-Light Anticancer Photodynamic Therapy
Source: Molecules. 2025 May 24;30(11):2310. doi: 10.3390/molecules30112310 (PMC12155855; doi:10.3390/molecules30112310)
Supplement: Supplementary file 1 [file molecules-30-02310-s001.zip › molecules-3590245-supplementary.pdf]

Push-pull OPEs in Blue-light Anticancer Photodynamic Therapy

Ana Lameiro,[a†] Chiara M.A. Gangemi,[b†] Aurora Mancuso,[b] Paola Maria Bonaccorsi,[b] Maria Letizia Di Pietro,[b] Silvia Gómez-Pastor,[a] Fausto Puntoriero,[b] Francisco Sanz-Rodríguez[a*] Anna Barattucci[b*]

[a]Departamento de Biología. Facultad de Ciencias, Universidad Autó noma deMadrid, 28049 Madrid, Spain.

francisco.sanz@uam.es

[b]Dipartimento di Scienze Chimiche, Biologiche, Farmaceutiche ed Ambientali (ChiBioFarAm), Universitàdegli Studi di Messina, 98166 Messina, Italy

abarattucci@unime.it

TABLE OF CONTEXT

Table S1	pag. S2
^{1}H, ^{13}C nmr spectra	pag. S3-S19
^{19}F nmr spectra of **OPE-NOF** and **OPE-ONF**	pag. S20-S21
Photostability	pag. S22
Normalized emission spectra of **OPE-NOF** in different solvents	pag. S23
Singlet oxygen luminescence	pag. S24
Raw data for ROS Quantum Yield	pag. S24
Drug and DMSO controls for HeLa cells	pag. S25
Toxicity data and IC_{50} values for **OPE-ONF** and **OPE-NOF**	pag. S26
Flow cytometry assays on **OPE-ONF**	pag. S27

Table S1: reaction conditions for the synthesis of 2

Entry	**A** (eq.)	**1** (eq.)	Catalyst	Solvent	time / T	Products
**A**	2	1	$Pd(PPh_3)_4$ (0.1 eq.)	DMF / Et_3N 1:1	24 h / 60 °C	**2 : 3** 1:3
**B**	3	1	$Pd(PPh_3)_2Cl_2$ (0.025 eq.) / CuI (0.025 eq.) / PPh_3 (0.05 eq.)	DMF / Et_3N 1:2	1 h / 65 °C	**2 : 3** 1:3
**C**	3	1	$Pd(PPh_3)_2Cl_2$ (0.025 eq.) / CuI (0.025 eq.)	DMF / Et_3N 1:2	1 h / 65 °C	**2 : 3** 1:4
**D**	3	1	$Pd(PPh_3)_2Cl_2$ (0.01 eq.) / CuI (0.01 eq.)	DMF / Et_3N 1:2	1 h / 65 °C	**2 : 3** 1:5
**E**	3	1	$Pd(PPh_3)_2Cl_2$ (0.025 eq.)	DMF / Et_3N 1:2	24 h / 80 °C	Almost only **3**
**F**	3	1	$Pd(PPh_3)_2Cl_2$ (0.025 eq.) / CuI (0.025 eq.)	DMF / Et_3N 1:2	24 h / rt	Almost only **3**
**G**	3	1	$Pd(PPh_3)_2Cl_2$ (0.025 eq.) / CuI (0.025 eq.) / PPh_3 (0.05 eq.)	DMF / Et_3N 1:2	24 h / rt	**1 : 2 : 3** 2:1:4
**H**	3	1	$Pd(PPh_3)_2Cl_2$ (0.025 eq.) / CuI (0.025 eq.)	$^iPr_2NH$	1 h / 65 °C	**2 : 3** 1:4
**I**	4	1	$Pd(PPh_3)_2Cl_2$ (0.025 eq.) / CuI (0.025 eq.) / PPh_3 (0.05 eq.)	Et_3N	1 h / 80 °C	**2 : 3** 8:1

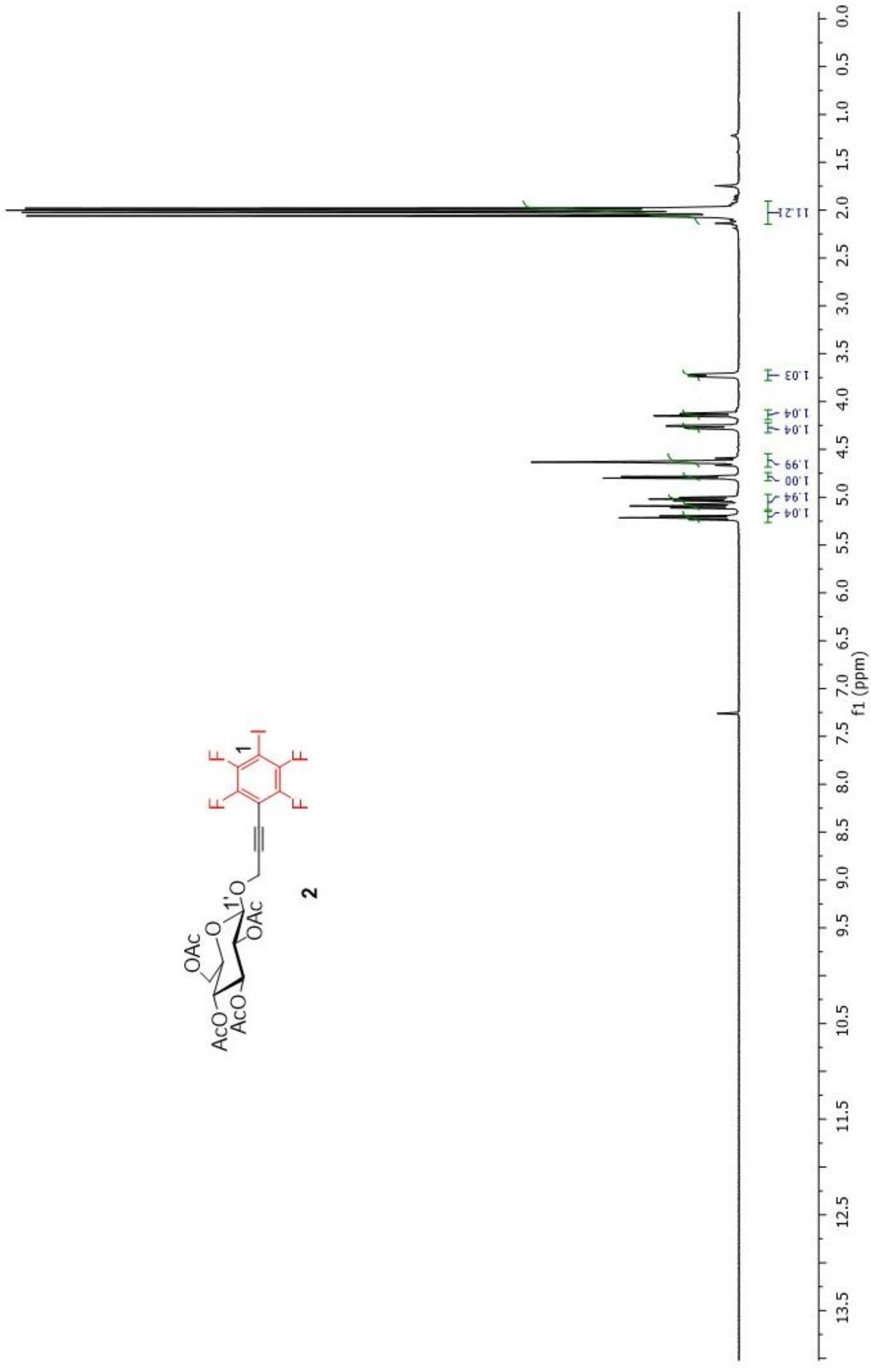

Figure **S1**: ^{1}H-NMR spectrum of compound **2** in $CDCl_3$

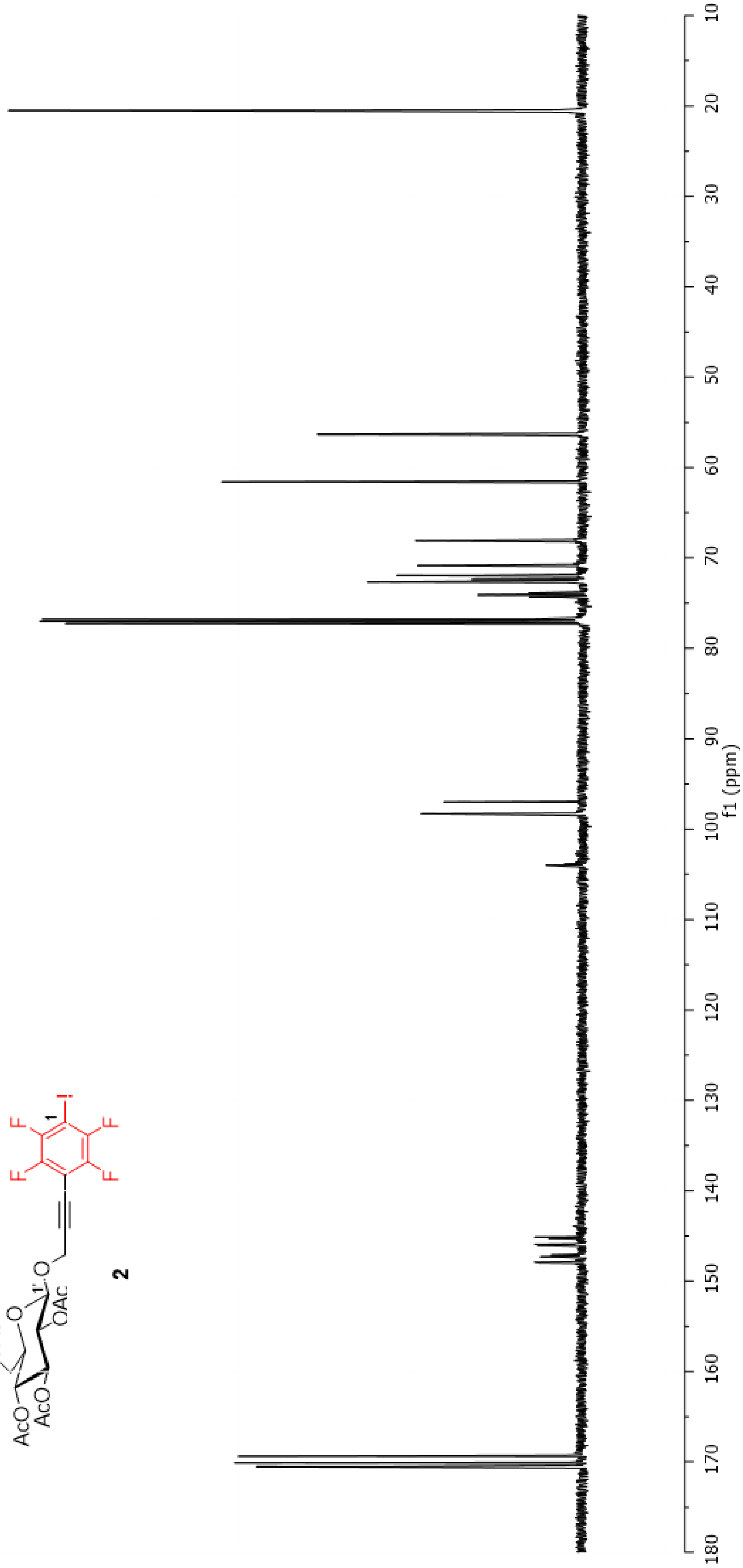

Figure **S2**: ^{13}C-NMR spectrum of compound **2** in $CDCl_3$

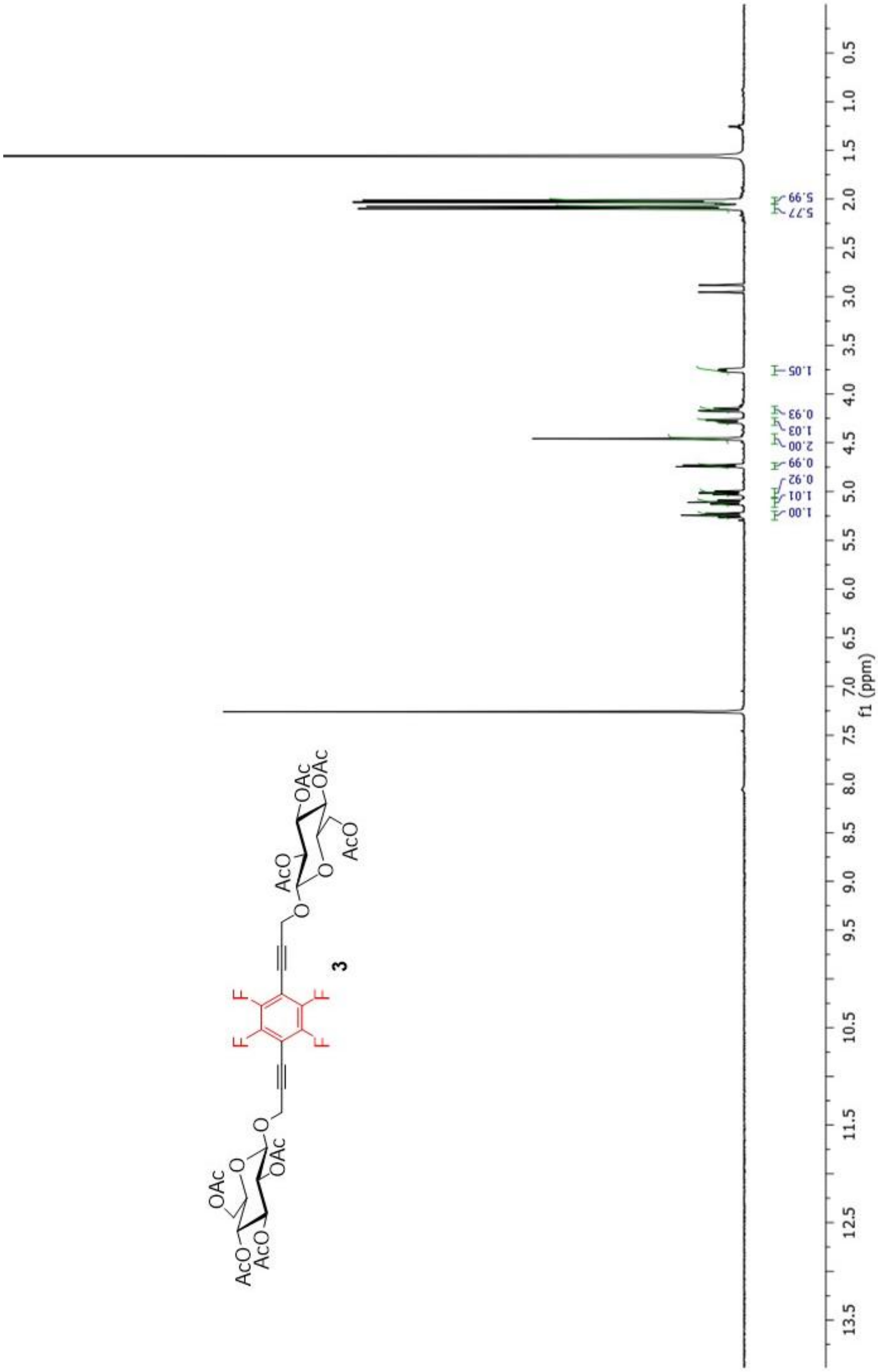

Figure **S3**: ^{1}H-NMR spectrum of compound **3** in $CDCl_3$

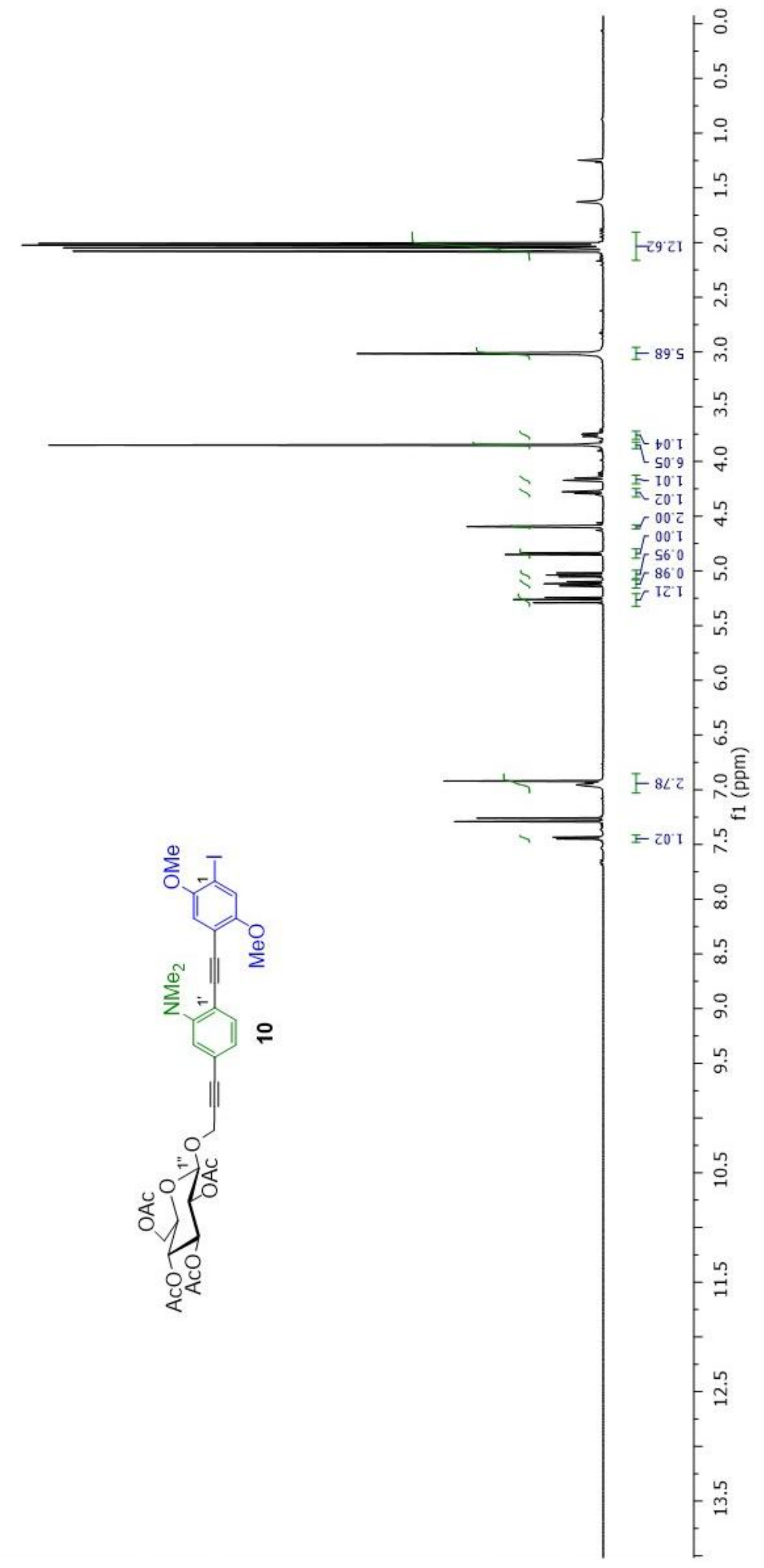

Figure **S4**: ^{1}H-NMR spectrum of compound **10** in $CDCl_3$

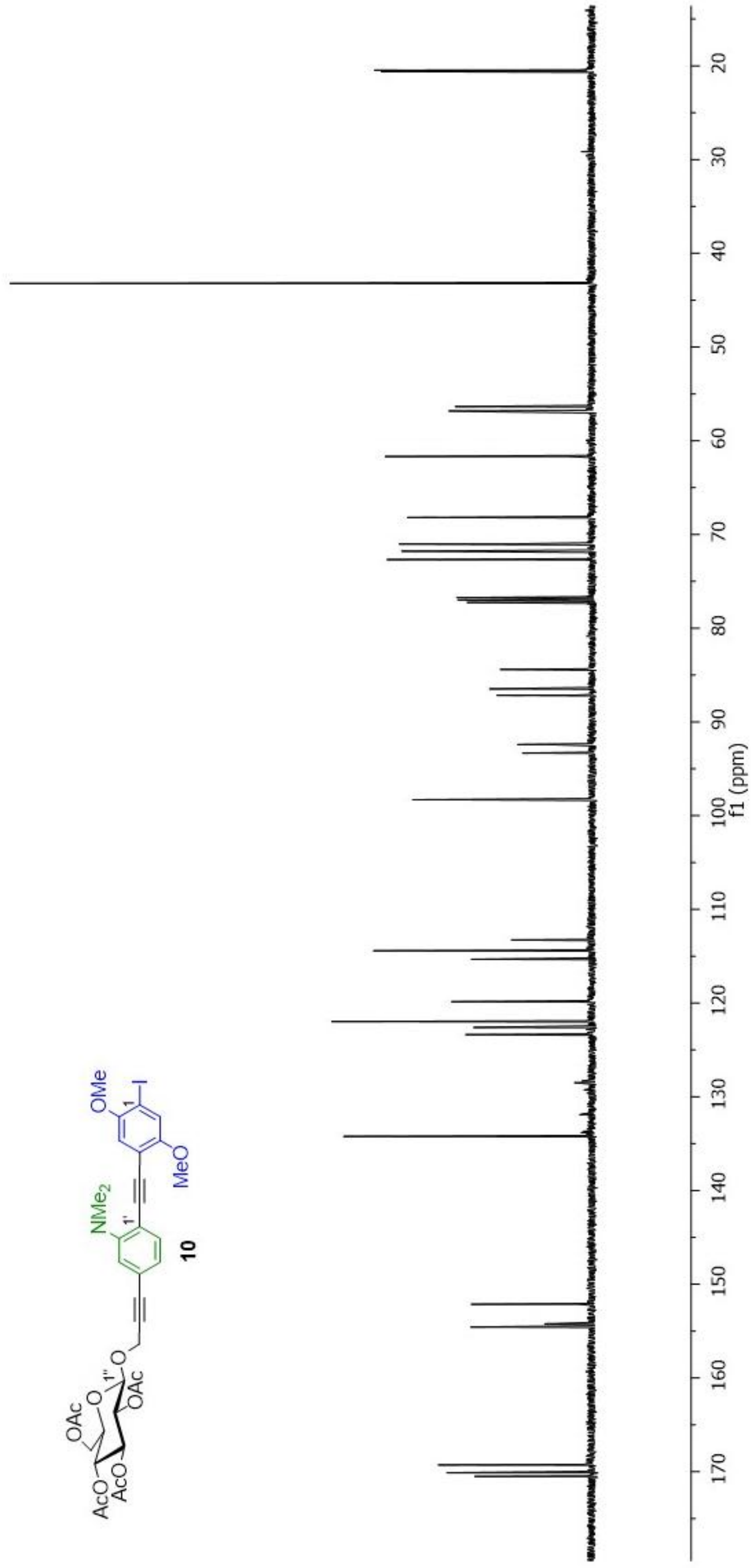

Figure **S5**: ^{13}C-NMR spectrum of compound **10** in $CDCl_3$

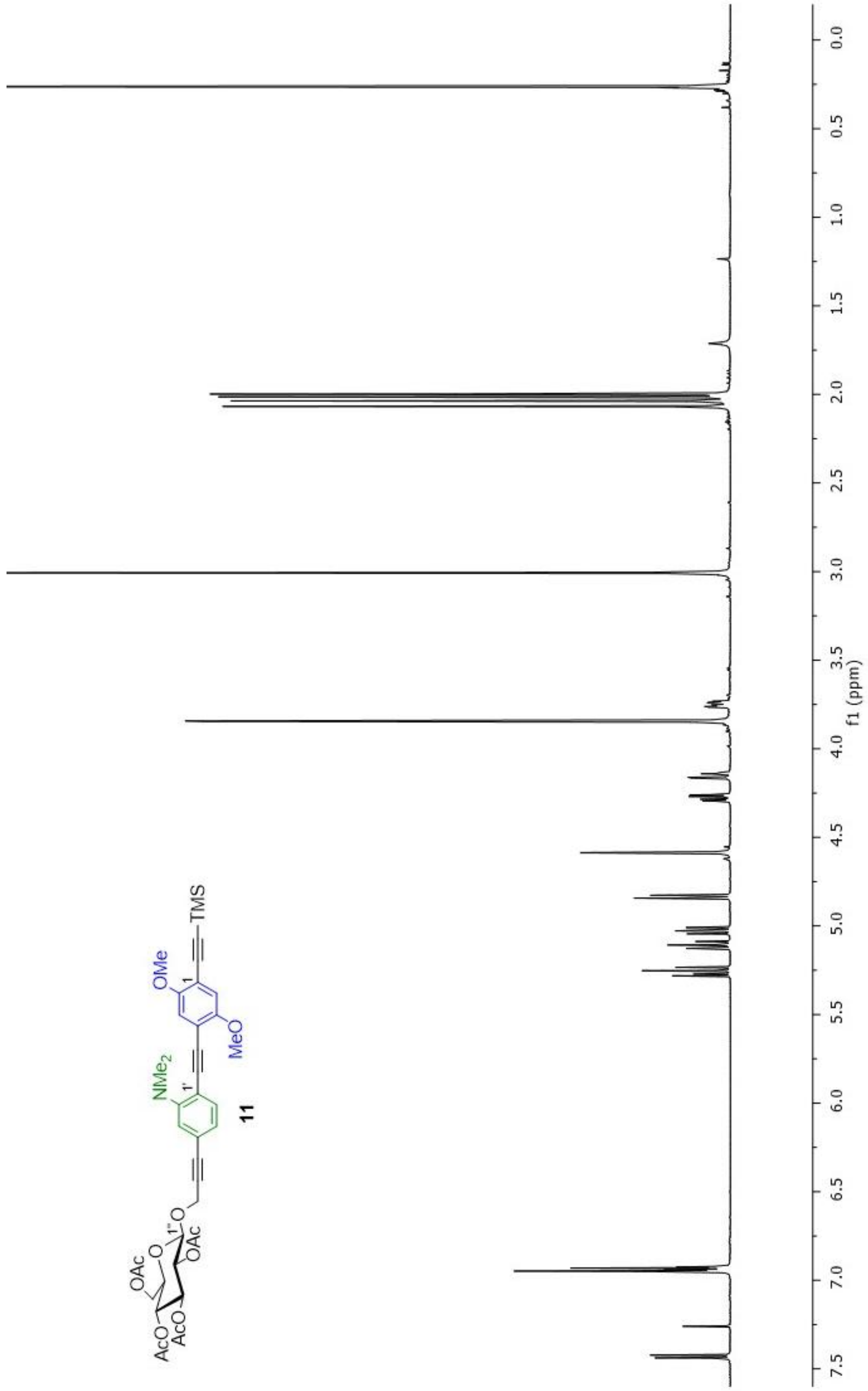

Figure **S6**: ^{1}H-NMR spectrum of compound **11** in $CDCl_3$

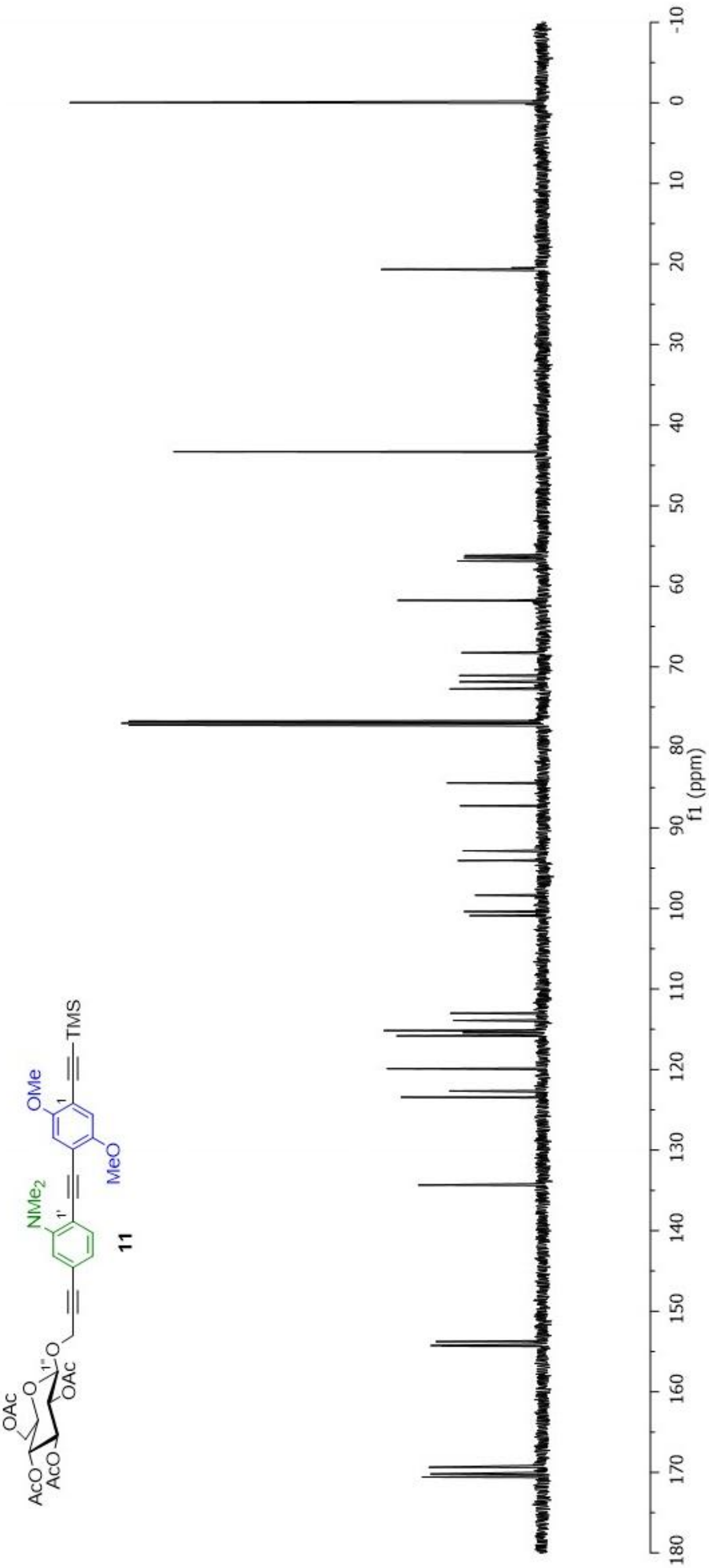

Figure **S7**: ^{13}C-NMR spectrum of compound **11** in $CDCl_3$

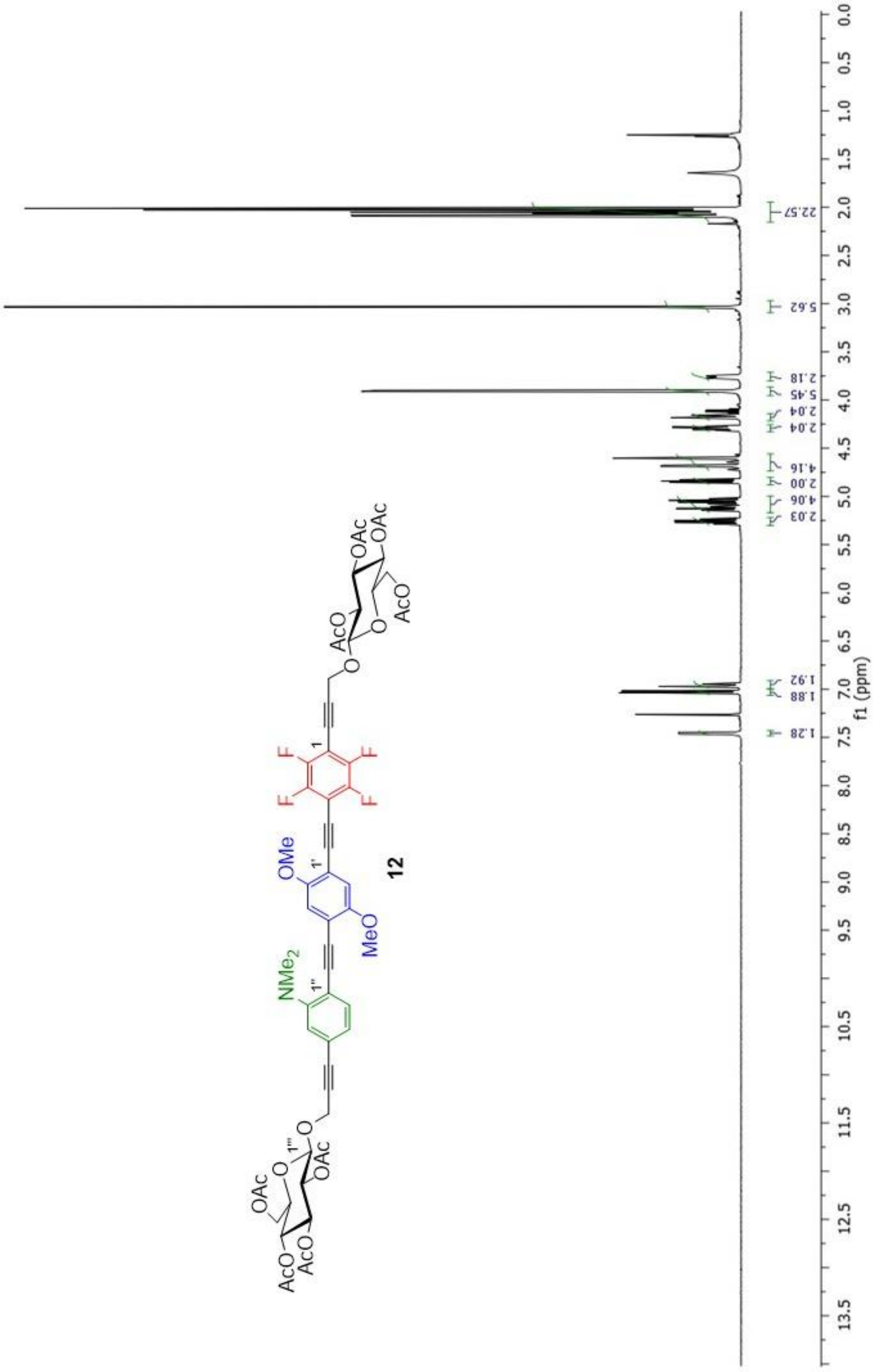

Figure **S8**: ^{1}H-NMR spectrum of compound **12** in $CDCl_3$

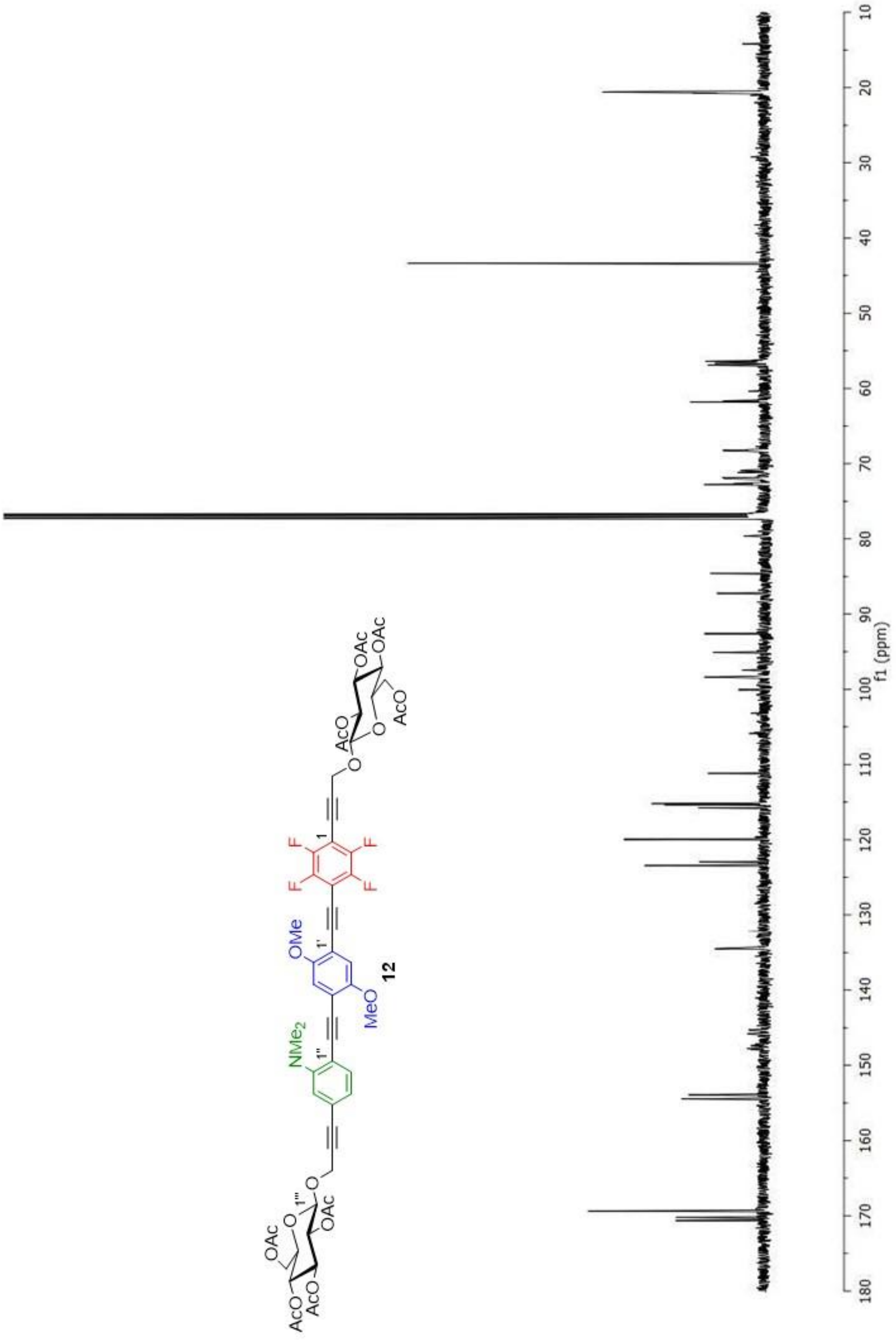

Figure **S9**: ^{13}C-NMR spectrum of compound **12** in $CDCl_3$

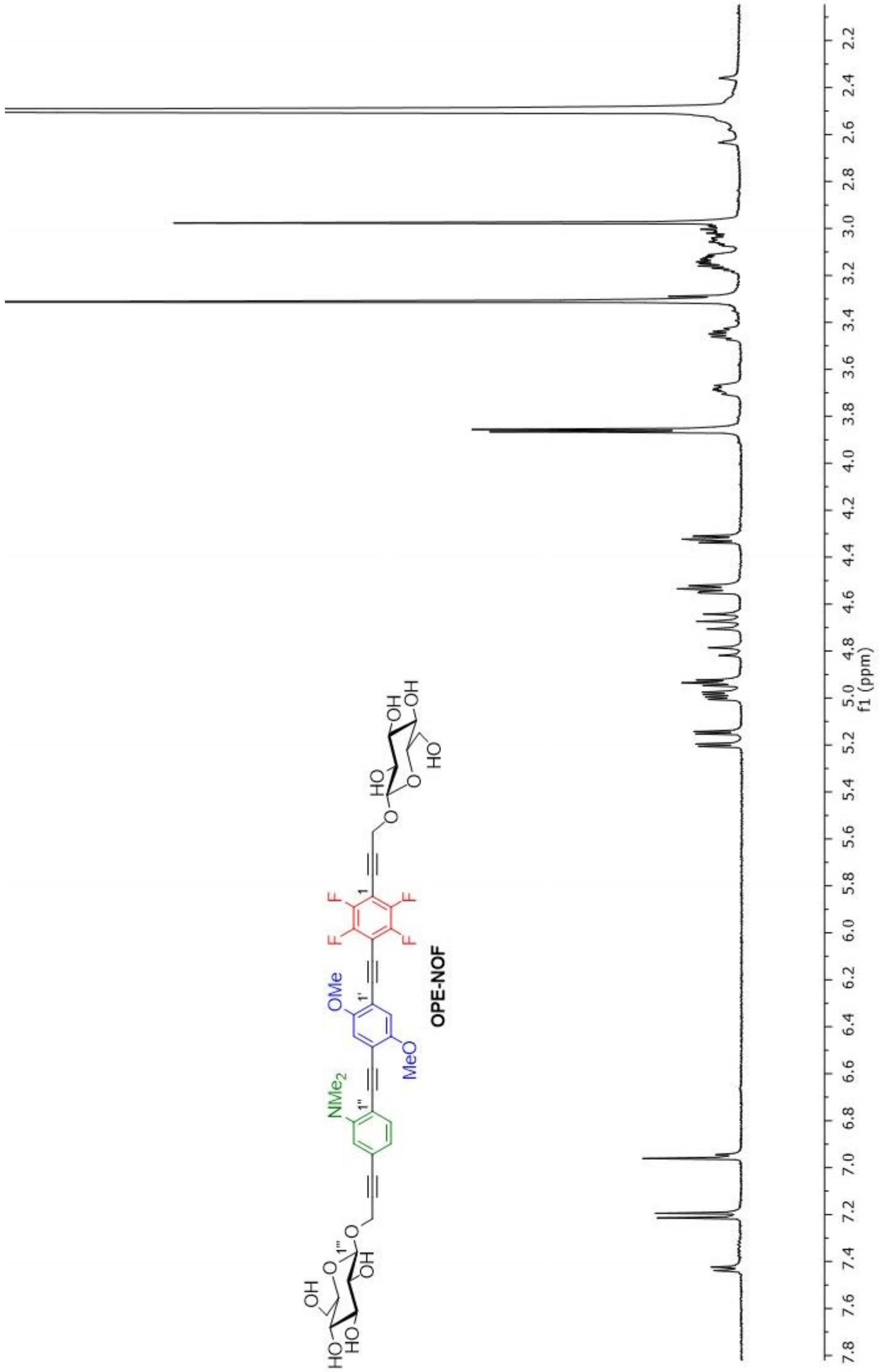

Figure **S10**: ^{1}H-NMR spectrum of compound **OPE-NOF** in DMSO-d_6

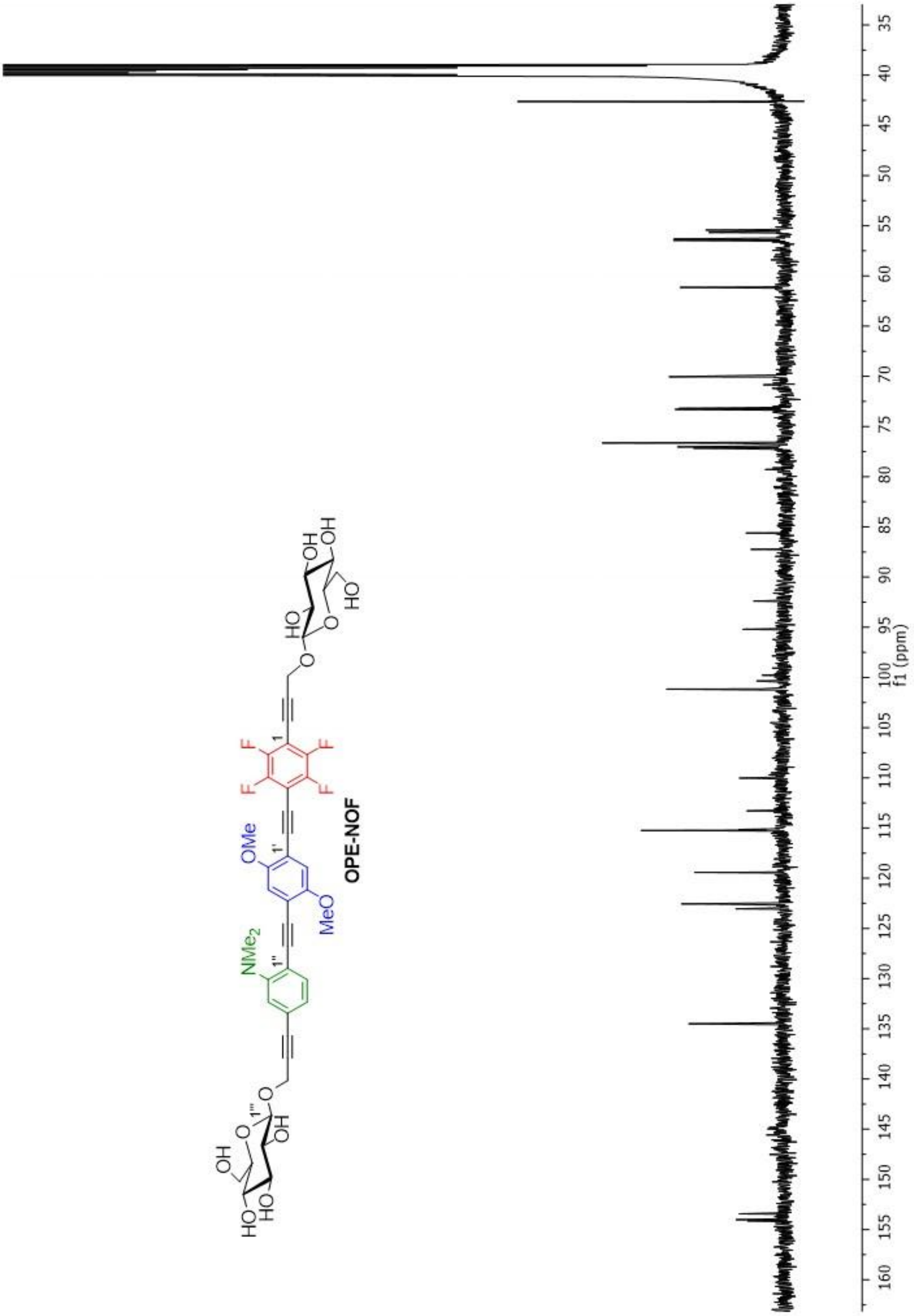

Figure **S11**: ^{13}C-NMR spectrum of compound **OPE-NOF** in DMSO-d_6

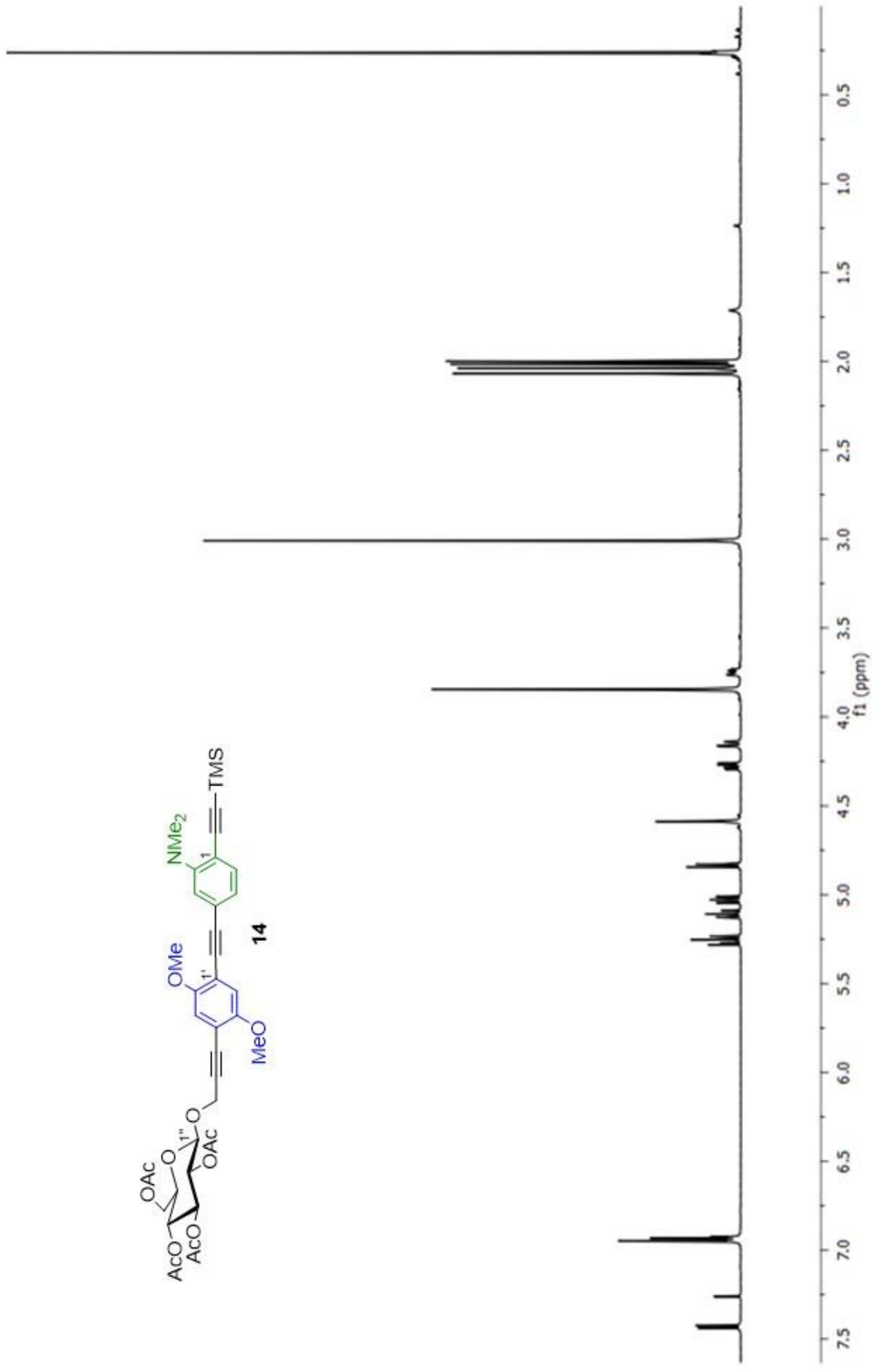

Figure **S12**: ^{1}H-NMR spectrum of compound **14** in $CDCl_3$

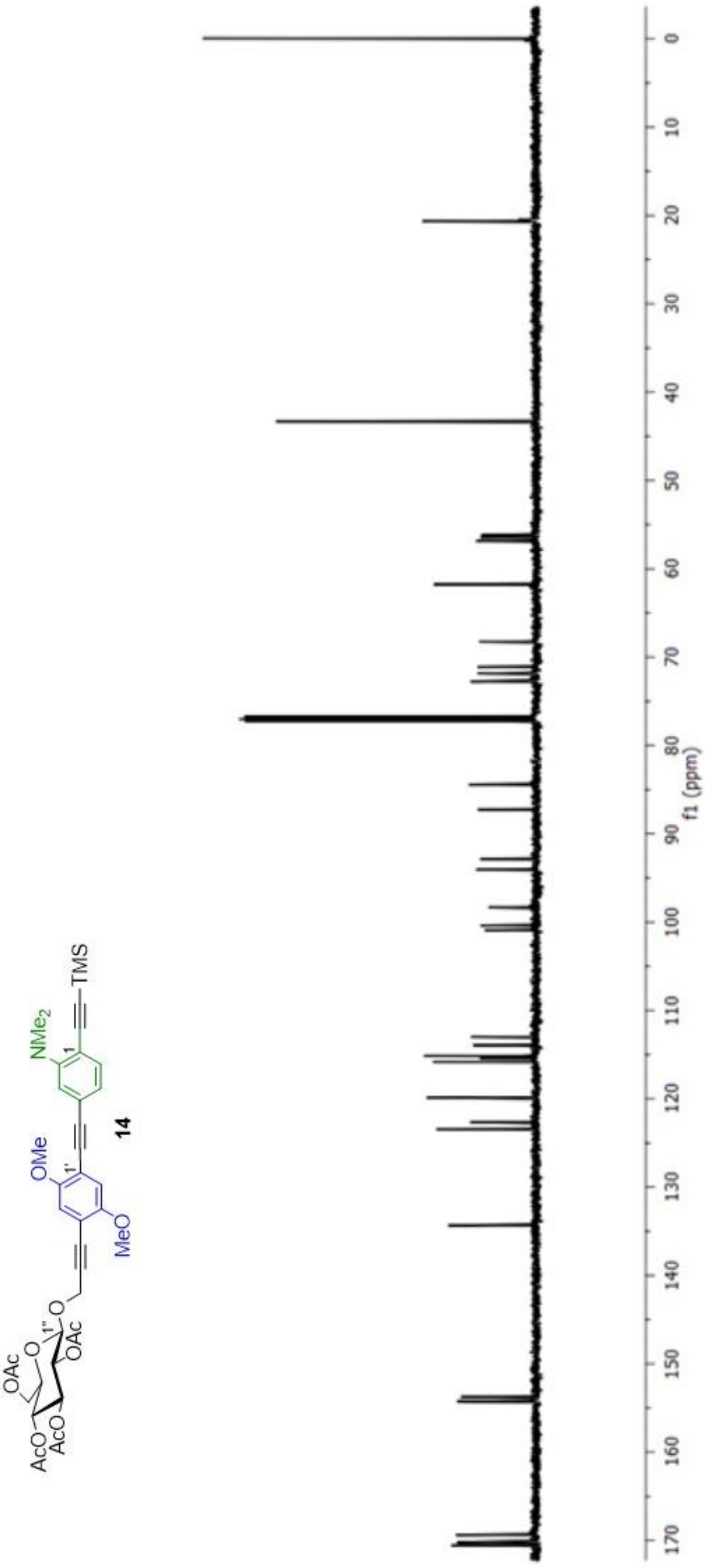

Figure **S13**: ^{13}C-NMR spectrum of compound **14** in $CDCl_3$

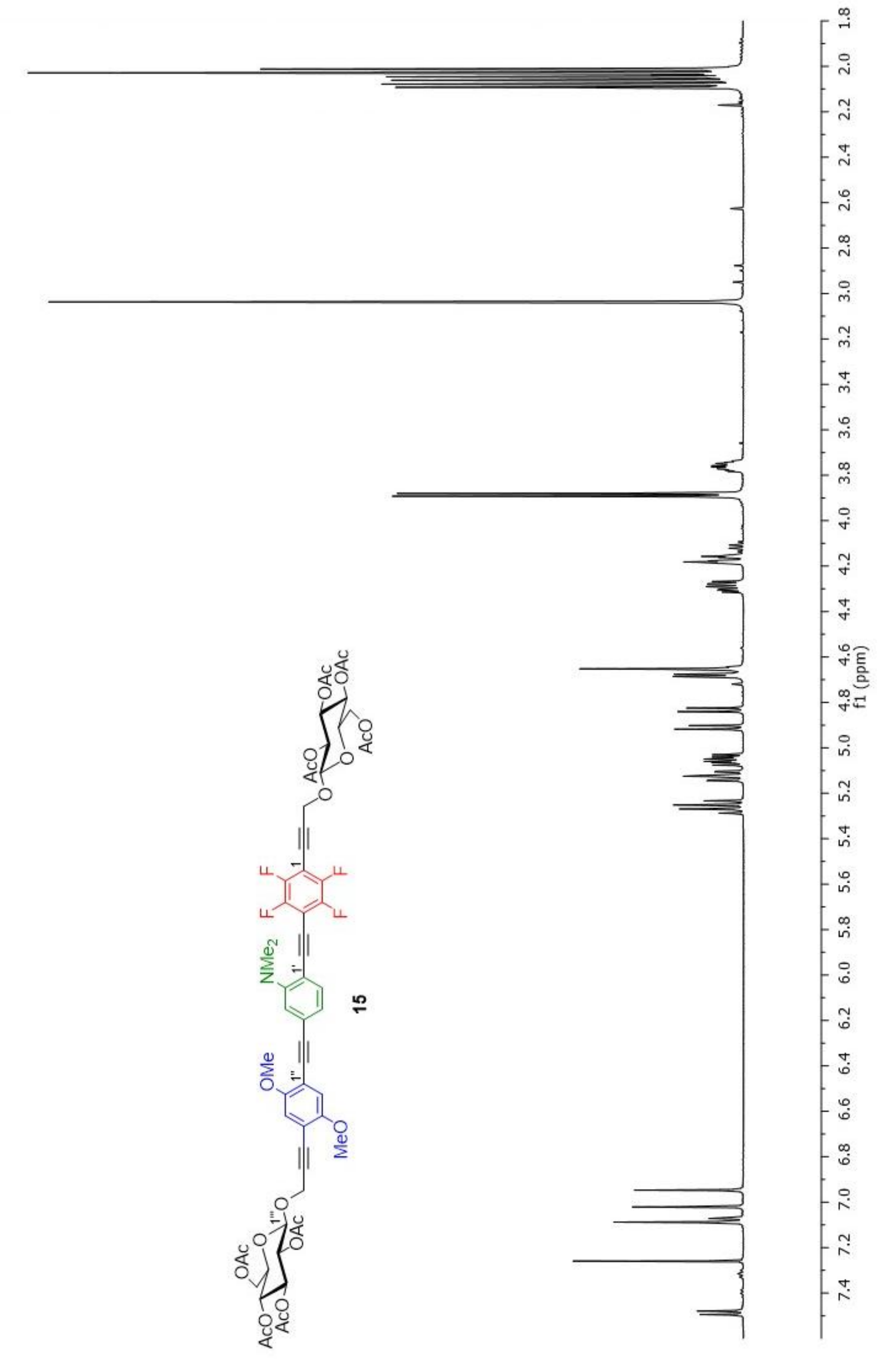

Figure **S14**: ^{1}H-NMR spectrum of compound **15** in $CDCl_3$

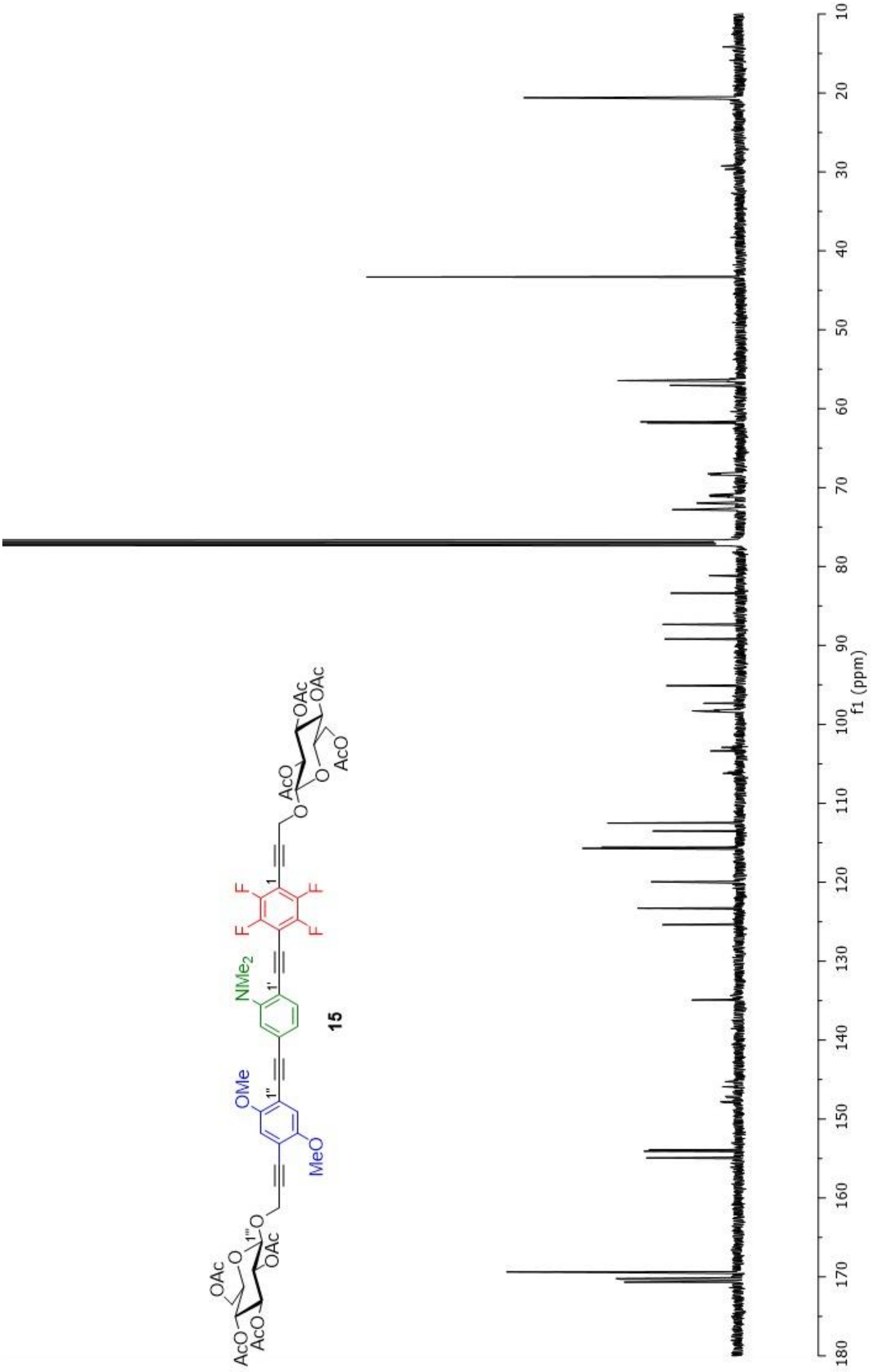

Figure **S15**: ^{13}C-NMR spectrum of compound **15** in $CDCl_3$

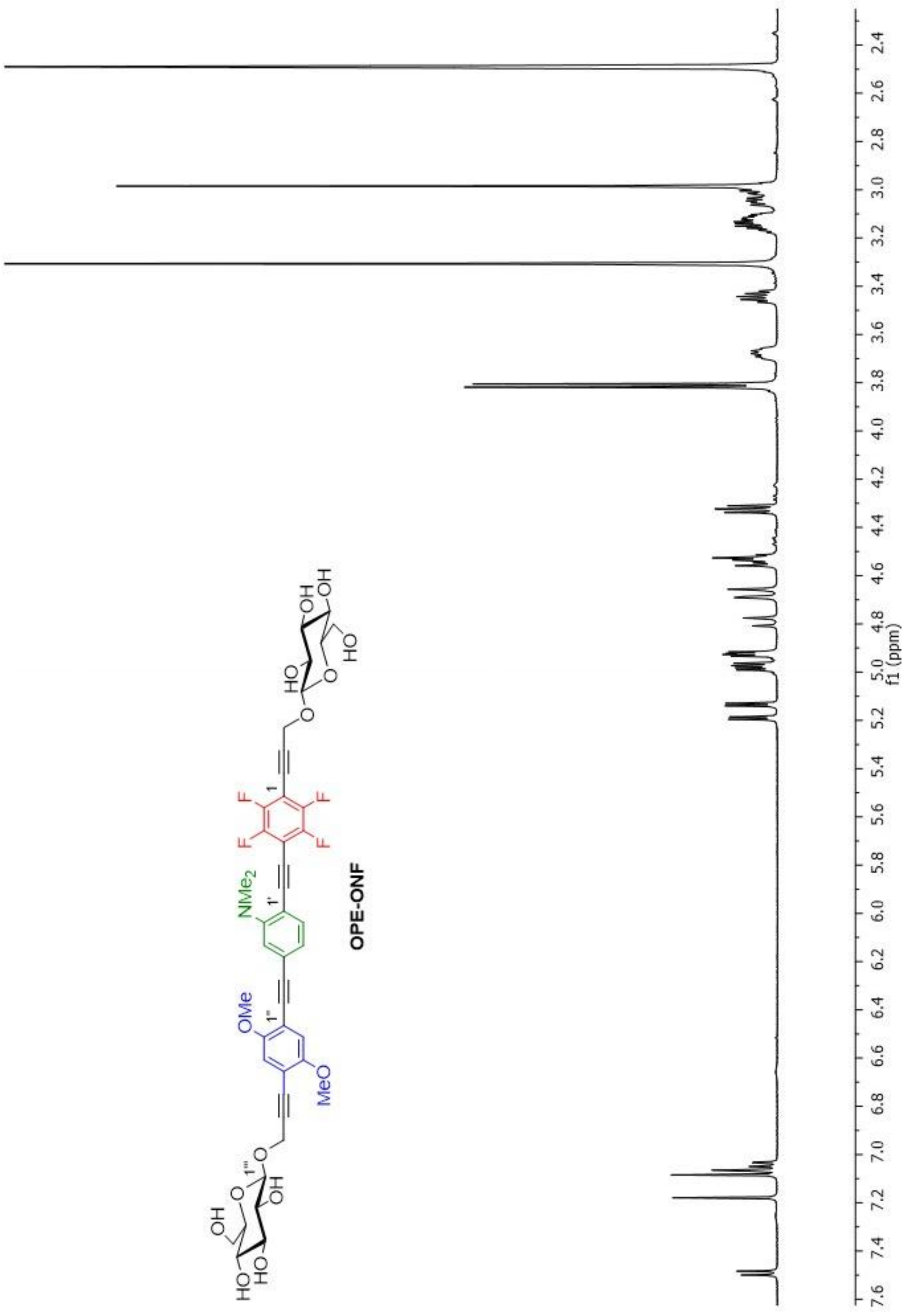

Figure **S16**: ^{1}H-NMR spectrum of compound **OPE-NOF** in DMSO-d_6

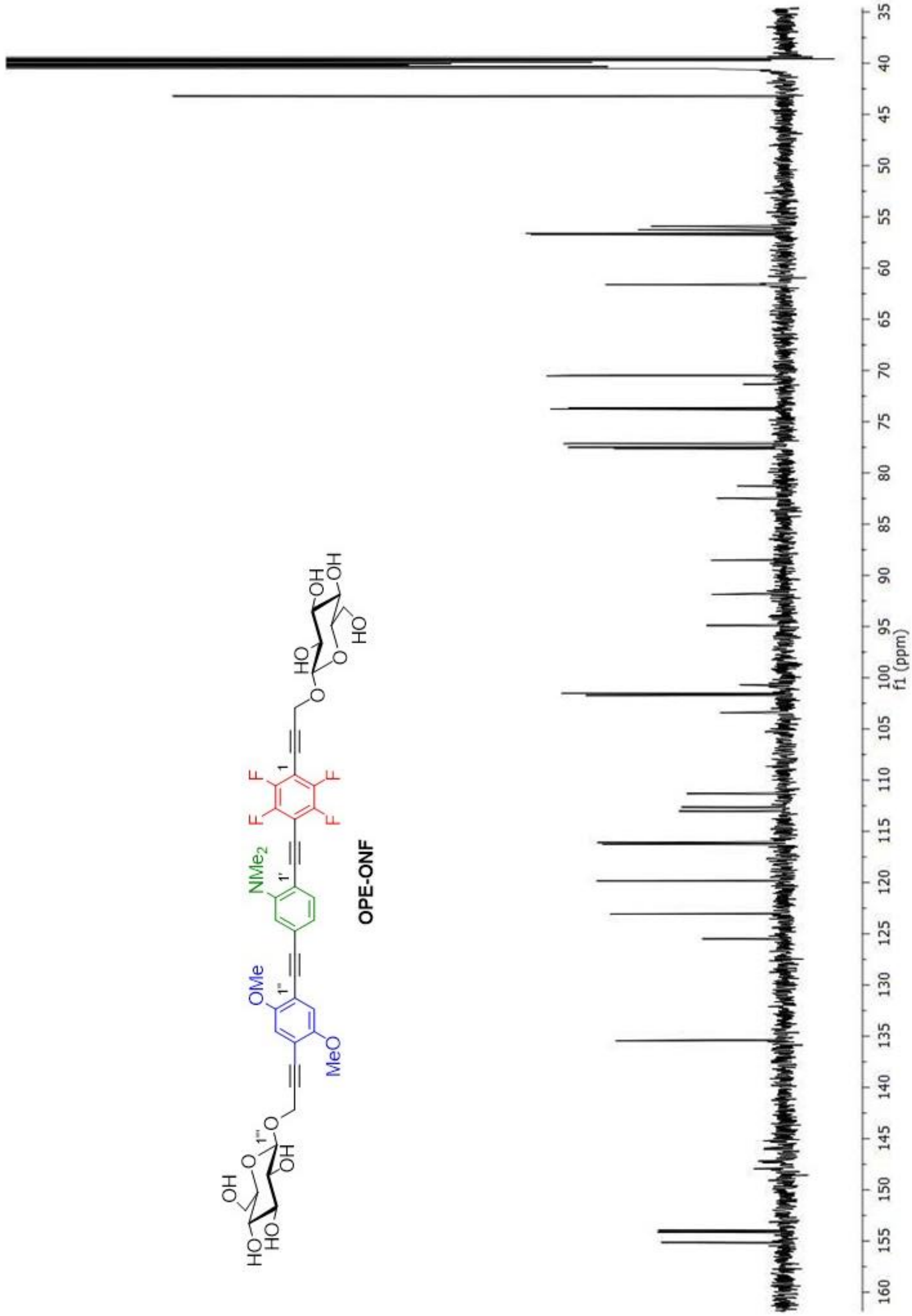

Figure **S17**: ^{13}C-NMR spectrum of compound **OPE-ONF** in DMSO-d_6

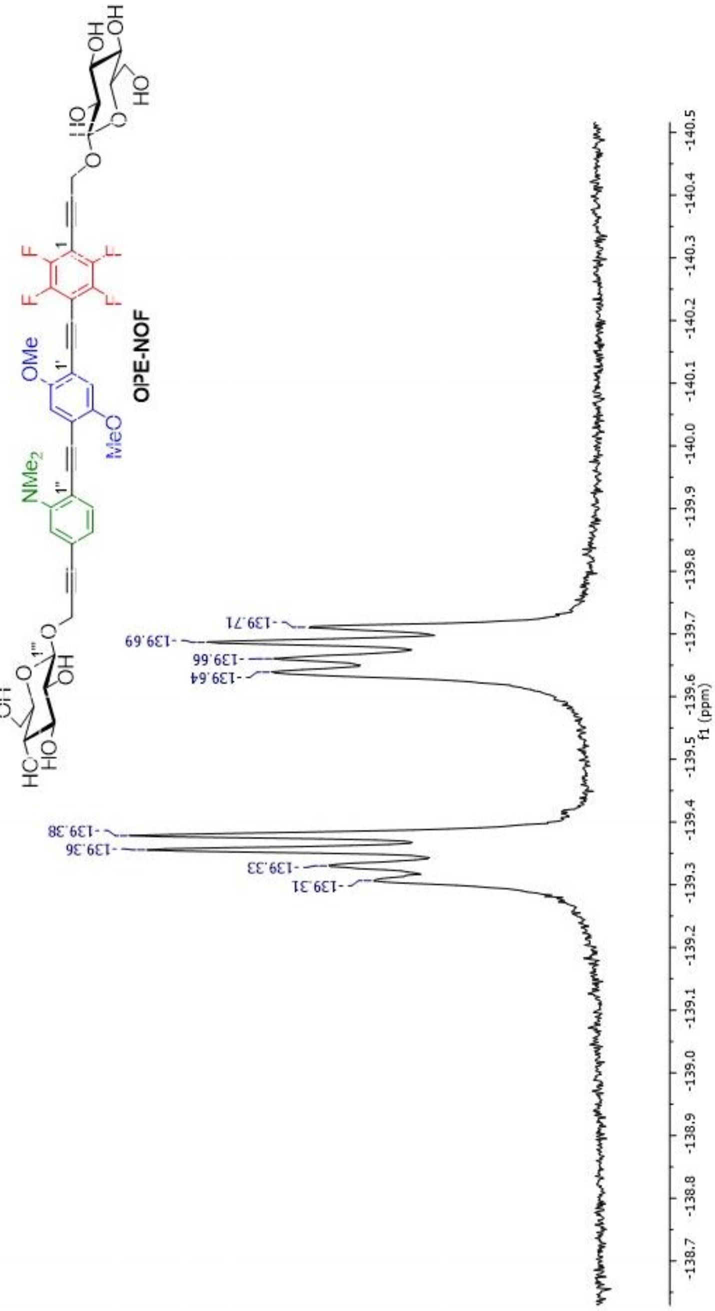

Figure **S18**:^{19}F-NMR spectrum of compound **OPE-NOF** in DMSO-d$_6$

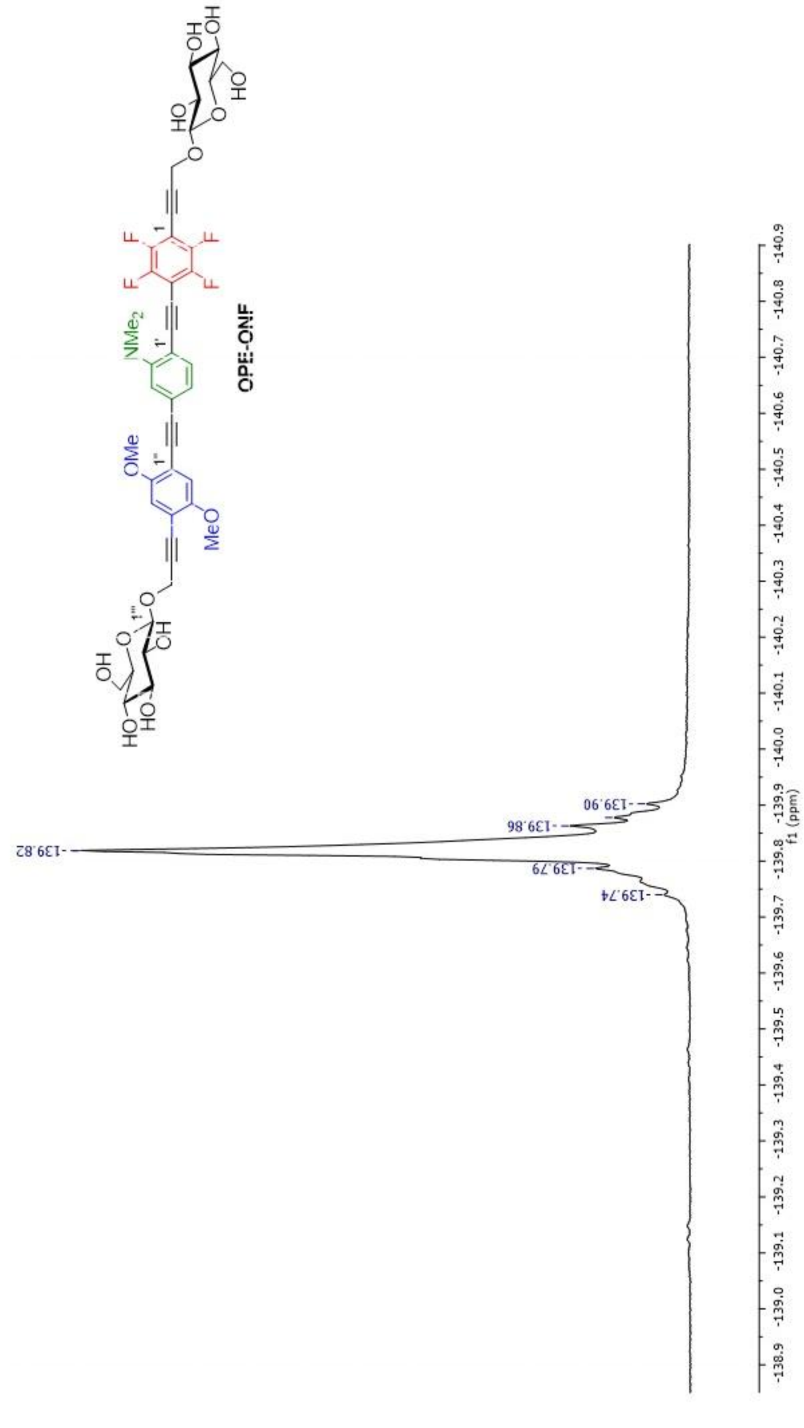

Figure **S19**:^{19}F-NMR spectrum of compound **OPE-ONF** in DMSO-d_6

- **Photostability**

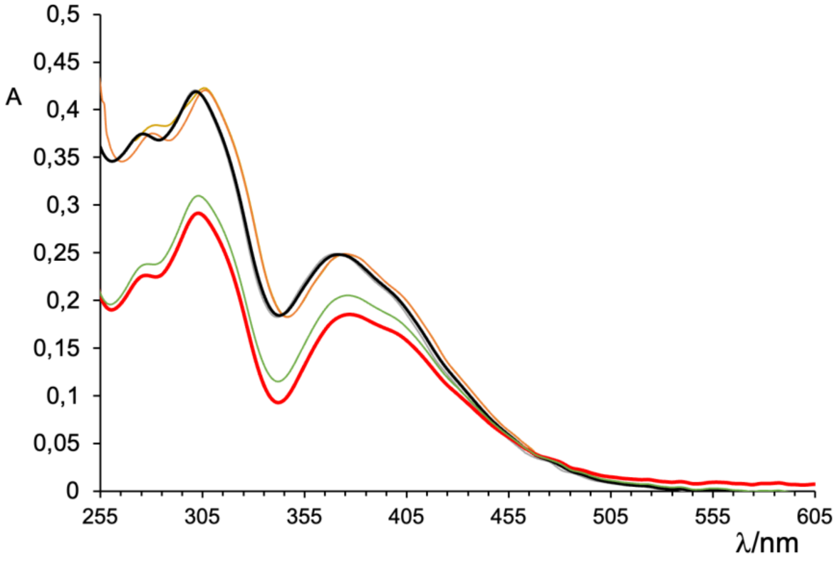

Figure S20. Absorption spectra of **OPE-NOF** in aqueous solution before irradiation (black line) and after 30 minutes (orange line), 1 hour (green line), and 1.5 hours (red line) of blue light irradiation at 450 nm.

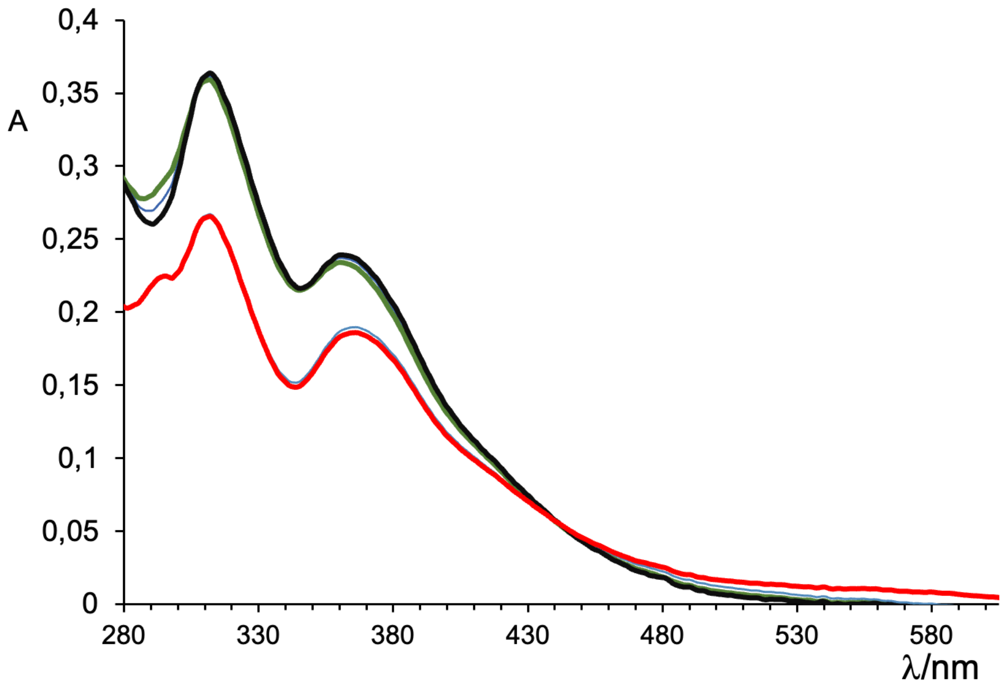

Figure S21. Absorption of **OPE-ONF** in aqueous solution before irradiation (black line) and after 30 minutes (green line), 1 hour (blue line), and 1.5 hours (red line) of blue light irradiation at 450 nm.

- **Solvents effect on luminescence**

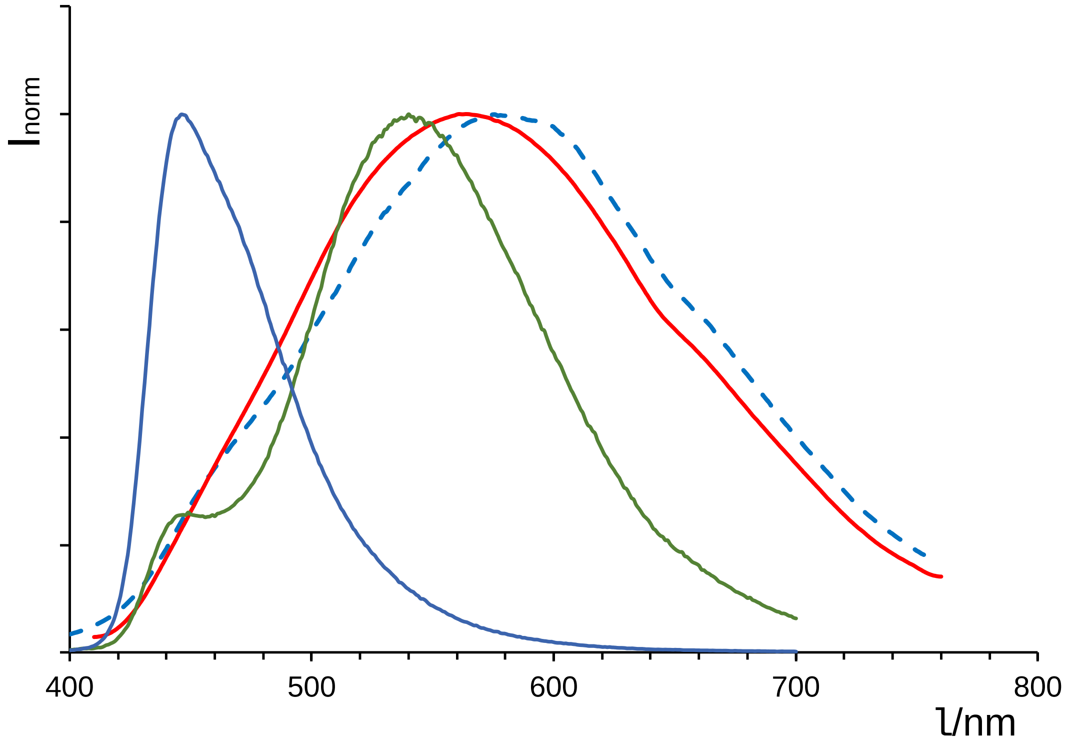

Figure S22. Normalized emission spectra of **OPE-NOF** in aqueous solution before (dashed blue line) and after the addition of one equivalent of acetic acid (solid blue line), in dichloromethane (green line), and in acetonitrile (red line). The emission in organic solvents is weak due to the poor solubility of **OPE-NOF** in these media.

- **Singlet oxygen luminescence**

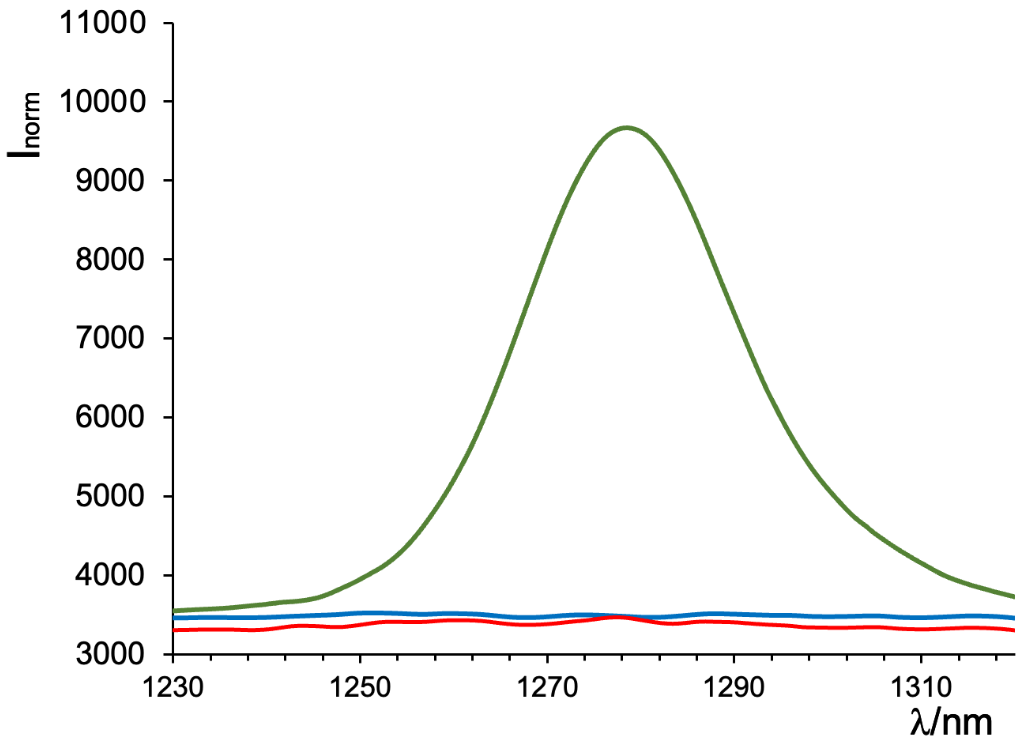

Figure S23. Emission spectra of **OPE-NOF** (blue line), **OPE-ONF** (orange line), and **TPP** (green line) in aqueous solution upon excitation at 450 nm.

- **Raw data for ROS Quantum Yield**

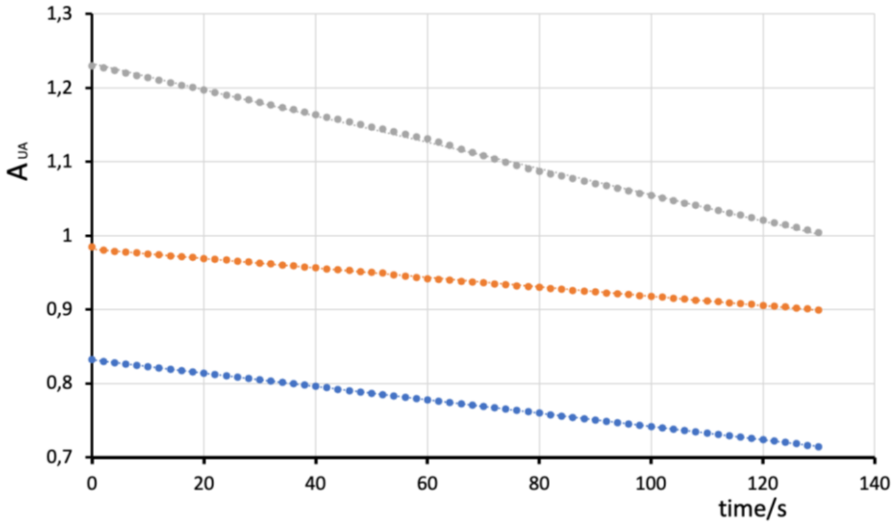

Figure S24. Absorbance trend at 280 nm at the absorption maximum of uric acid upon irradiation of an aqueous solution of **OPE-ONF** (orange) and **OPE-NOF** (blue), both isoabsorbing at 450 nm. The degradation profile of uric acid in the presence of methylene blue irradiated under the same photon flux conditions is shown in gray. The data were used to estimate the quantum yield of ROS production.

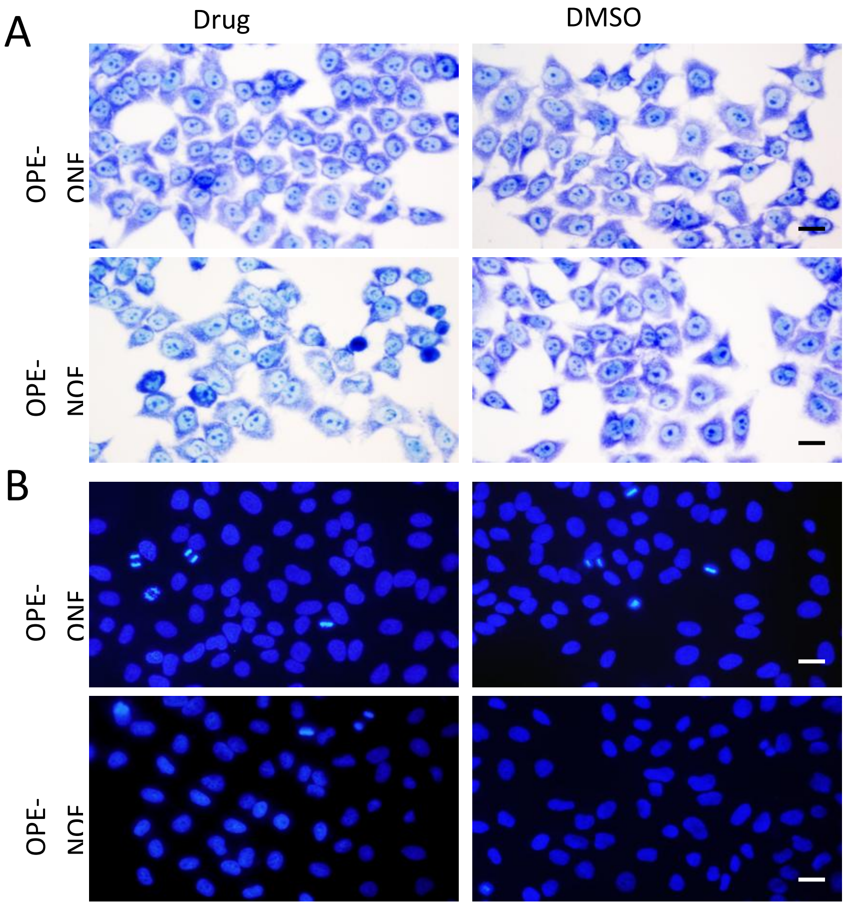

Figure S25. Drug and DMSO controls for HeLa cells subjected to blue light PDT with **OPE-ONF** and **OPE-NOF**. (A) HeLa cells were incubated for 2 hours with 10^{-5} M **OPE-ONF** or **OPE-NOF**. In parallel, cells were treated with an equivalent volume of DMSO corresponding to that used in compound-treated samples. After 24 hours, cells were stained with toluidine blue and observed under light microscopy. (B) HeLa cells were incubated for 2 hours with 10^{-5} M **OPE-ONF** or **OPE-NOF**. In parallel, cells were treated with an equivalent volume of DMSO corresponding to that used in compound-treated samples. After 24 hours, cells were stained with DAPI and observed under fluorescence microscopy. Scale bar: 20 µm.

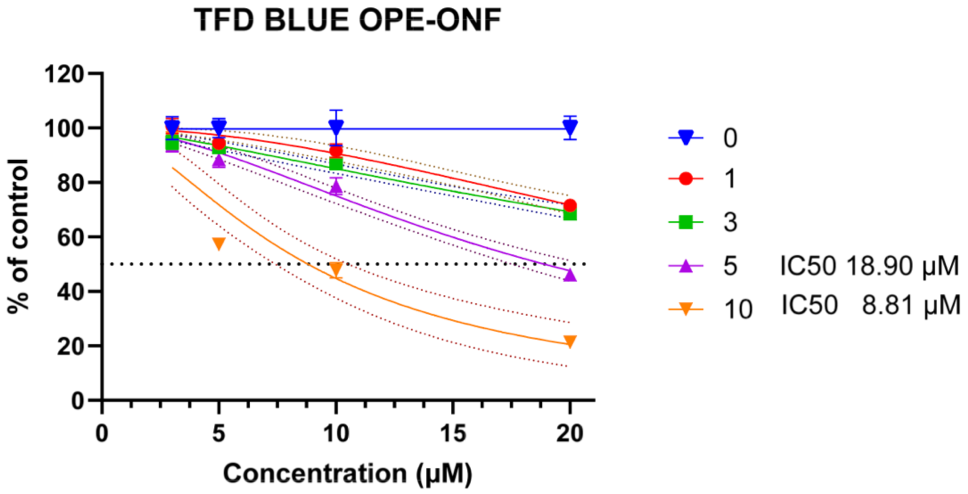

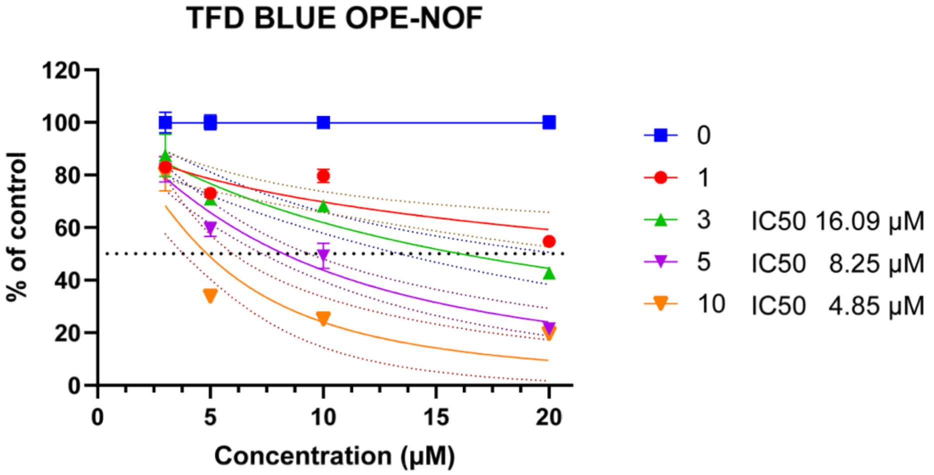

Figure S26: Toxicity data and IC_{50} values for **OPE-ONF** and **OPE-NOF** after irradiation with blue light for 0, 1, 3, 5, 10 minutes.

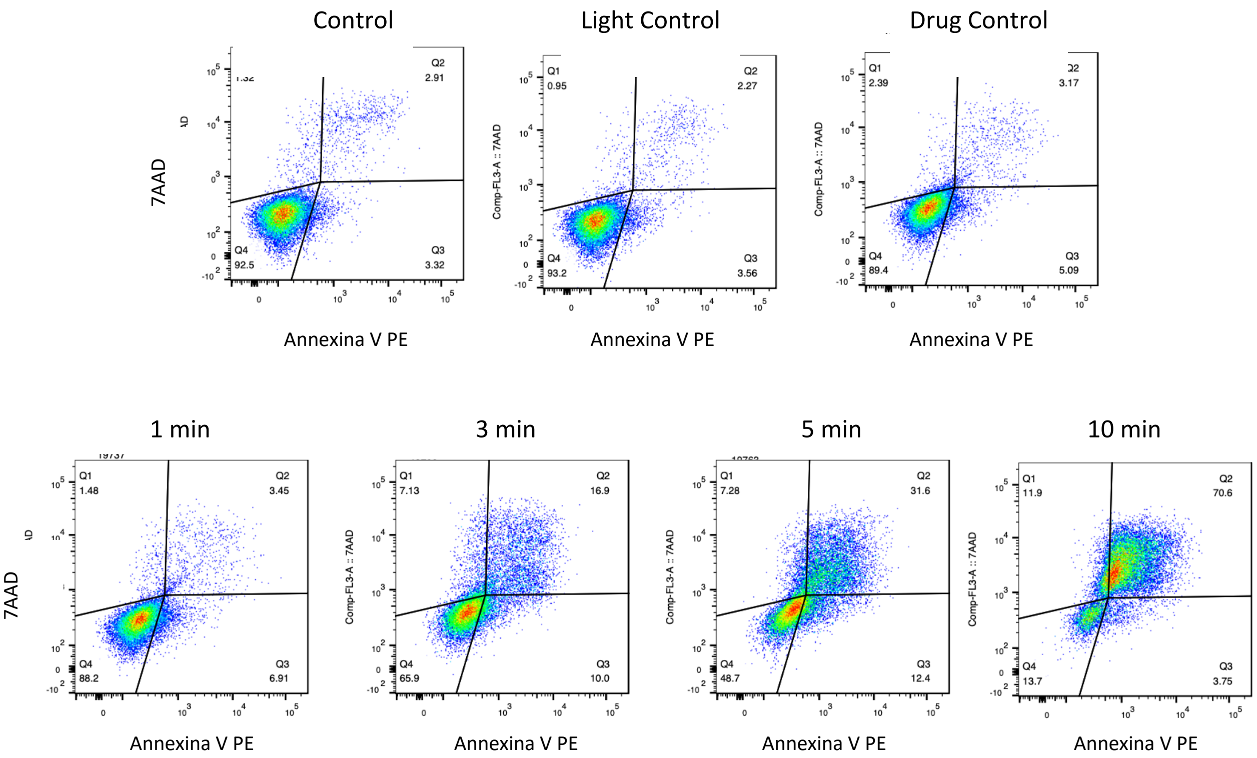

Figure S27. Flow cytometry assays on **OPE- ONF**[1] using the PE Annexin V Apoptosis Detection Kit with 7-AAD. HeLa cells were incubated with 1 x 10-5 M **OPE-ONF** and irradiated 1, 3, 5 and 10 minutes with blue light (450 nm). Following photodynamic treatment with **OPE-ONF**, we observed a time-dependent increase in the percentage of cells undergoing late apoptosis (Q2 quadrant), rising from 3.45% after 1 minute of irradiation to 70.6% after 10 minutes. The marked increase in late apoptotic cells upon treatment with **OPE-ONF** suggests compromised membrane integrity; however, this precludes a definitive conclusion as to whether the cell death is apoptotic or necrotic in nature. These findings are consistent with our morphological analysis, which also supports the interpretation that **OPE-ONF** and **OPE-NOF** primarily induce necrotic cell death.

[1] We focused on **OPE-ONF** for these experiments, as its photodynamic effect is comparatively less aggressive, and the resulting cell death features may be more ambiguous, potentially leading to misinterpretation between apoptotic and necrotic pathways
